# Cigarette Smoke Triggers Loss of Corneal Endothelial Cells and Disruption of Descemet's Membrane Proteins in Mice

**DOI:** 10.1167/iovs.62.3.3

**Published:** 2021-03-02

**Authors:** Muhammad Ali, Shahid Y. Khan, Yura Jang, Chan Hyun Na, C. Conover Talbot, John D. Gottsch, James T. Handa, S. Amer Riazuddin

**Affiliations:** 1 The Wilmer Eye Institute, Johns Hopkins University School of Medicine, Baltimore, Maryland, United States; 2 Department of Neurology, Johns Hopkins University School of Medicine, Baltimore, Maryland, United States; 3 Institute for Basic Biomedical Sciences, Johns Hopkins University School of Medicine, Baltimore, Maryland, United States

**Keywords:** corneal endothelium, proteome, cigarette smoke

## Abstract

**Purpose:**

To investigate changes at a molecular level in the mouse corneal endothelium (CE) exposed to chronic cigarette smoke (CS).

**Methods:**

Pregnant mice (gestation days 18–20) were placed in a whole-body exposure smoking chamber, and a few days later pups were born. After 3.5 months of CS exposure, a ConfoScan4 scanning microscope was used to examine the corneal endothelial cells (CECs) of CS-exposed and control (Ct) mice. The CE was peeled under a microscope and maintained as four biological replicates (two male and two female) for CS-exposed and Ct mice; each replicate consisted of 16 CEs. The proteome of the CE was investigated through mass spectrometry.

**Results:**

The CE images of CS-exposed and Ct mice revealed a difference in the shape of CECs accompanied by a nearly 10% decrease in CEC density (*P* < 0.00003) following CS exposure. Proteome profiling identified a total of 524 proteins exhibiting statistically significant changes in CE from CS-exposed mice. Importantly, proteins associated with Descemet's membrane (DM), including COL4α1, COL4α2, COL4α3, COL4α4, COL4α5, COL4α6, COL8α1, COL8α2, and FN1, among others, exhibited diminished protein levels in the CE of CS-exposed mice.

**Conclusions:**

Our data confirm that exposure to CS results in reduced CEC density accompanied by diminished levels of multiple collagen and extracellular matrix proteins associated with DM.

The cornea, an avascular tissue, consists of five anatomical distinctive layers: epithelium, Bowman's membrane, stroma, Descemet's membrane (DM), and endothelium.^1^ The corneal endothelium (CE), the most posterior layer of the cornea, consists of a monolayer of hexagonal-shaped endothelial cells that principally maintain corneal transparency.^2^ Because corneal endothelial cells (CECs) have limited proliferative potential, multiple factors, including endothelial dystrophies, surgical trauma, age, and smoking, contribute to CE cell loss and decreased CE cell density.

Cigarette smoke (CS) is a complex mixture of toxins (e.g., nicotine, carbon monoxide) that can affect the whole body.^3^^,^^4^ CS exposure has been linked to multiple ocular diseases, such as glaucoma, cataracts, and age-related macular degeneration.^5^^–^^7^ Multiple studies have shown the link between CS and corneal endothelial dystrophy, characterized by progressive CEC loss and resulting in corneal edema and guttae formation leading to vision loss.^8^^,^^9^ Kara and colleagues^10^ reported that CS affects the tear break-up time, but no significant differences in the mean central corneal thickness (CCT), mean CEC density, or parameters of endothelial cell morphology were observed between smokers and non-smokers.^10^ In another study, Golabchi and colleagues^11^ documented that smokers with severe nicotine dependency had substantially greater average cell size and lower CEC density as compared to non-smokers.

CS enhances the generation of free radicals and reactive oxygen species (ROS),^3^^,^^4^ and multiple investigations have shown susceptibility of CECs to hypoxia and oxidative stress.^12^^–^^16^ Even though long-term exposure to CS has been associated with many ocular disorders in humans,^17^^,^^18^ to date only a few studies evaluating the effects of CS on CECs have been reported,^19^^,^^20^ and the pathomechanism of this association remains elusive.

We investigated changes at a molecular level in mouse CE exposed to chronic CS, and our data confirm that exposure to CS results in reduced CEC density accompanied by diminished levels of multiple collagen and extracellular matrix (ECM) proteins associated with DM.

## Methods

### Animals Included in the Study

The use of mice in this study was approved by The Johns Hopkins Animal Care and Use Committee, and all experiments were performed in accordance with the approved protocol consistent with the ARVO statement for the Use of Animals in Ophthalmic and Vision Research. The protocol for this study was approved by the Institutional Review Board of The Johns Hopkins University School of Medicine. C57BL/6J mice (stock #000664; The Jackson Laboratory, Bar Harbor, ME, USA) were used for all experiments.

The mice were exposed to CS as described previously.^21^ The study design included placing four 14-week-old pregnant mice (gestation days 18–20) in a whole-body exposure smoking chamber, and a few days later pups were born. Of these, four adult females along with 32 pups (16 male and 16 female) were exposed to CS for 5 hour/day, 5 day/week, and they remained in the chamber for a total of 105 days (3.5 months). In parallel, four adult females and 32 pups (16 male and 16 female) were housed at The Johns Hopkins animal facility for 3.5 months and served as the control (Ct). It is important to note that we used age-matched adult female mice to serve as a control to remain consistent with the age of the CS-exposed mice.

### Exposure to CS in a Smoke Chamber

The smoke chamber contained a smoking machine (TE-10, Teague Enterprises, Woodland, CA, USA) that burned five cigarettes (2R4F reference cigarette, 2.45 mg nicotine per cigarette; Tobacco Research Institute, University of Kentucky, Lexington, KY, USA) at a time, taking puffs of 2-second duration at a flow rate of 1.05 L/min, to provide a standard puff of 35 cm^3^, providing a total of eight puffs per minute. The machine was adjusted to produce sidestream (89%) and mainstream (11%) smoke. The chamber atmosphere was monitored to maintain total suspended particulates at 90 mg/m^3^ and carbon monoxide at 350 ppm.

### Evaluation of CE Phenotype in Mice Exposed to CS

The CE was examined after CS exposure (i.e., 105 days), along with the CE of the age-matched Ct mice. The eyes were dilated using topical administration of tropicamide (1%) and phenylephrine (2.5%) solutions. The mice were anesthetized by subcutaneous injection of ketamine/xylazine (100 mg/kg body weight for ketamine and 16 mg/kg body weight for xylazine), and the CE was examined using a ConfoScan4 (Nidek Technologies, Aichi, Japan) as described previously.^22^ The CE of both CS-exposed and Ct mice was examined using a ConfoScan4 scanning microscope, and CE imaging took 12 to 15 minutes per mouse, bilaterally. The pupils were dilated before imaging by applying eye drops (i.e., 1% tropicamide and 2.5% phenylephrine). Although, ConfoScan4 imaging of both CS-exposed and Ct mice was performed on two different days, mice in both groups were of the same age (i.e., postnatal day 105).

### Extraction of the CE

Immediately after the ConfoScan4 imaging, the mice were euthanized, the eyes were enucleated, and the CE was peeled from mouse corneas under a microscope. The extracted tissue from CS-exposed and Ct mice was maintained at −80°C in distinct pools (i.e., biological replicates). We used four biological replicates (two male and two female) of the CS-exposed and Ct mice; each of the four biological replicates consisted of 16 CEs (both right and left eyes) from four male or four female mice.

### Mass Spectrometry-Based Proteome Profiling

The proteomes of the CE extracted from the CS-exposed and Ct mice were investigated through mass spectrometry-based isobaric tandem mass tag (TMT) proteome profiling. Briefly, four biological replicates, each consisting of pooled CE isolated from the right and left eyes of eight male or female CS-exposed and Ct mice were used for an eight-plex TMT experiment. Sample processing, including protein isolation, digestion, labeling with TMT, and analysis using liquid chromatography with tandem mass spectrometry (MS/MS), was performed as described previously.^23^

Briefly, the frozen samples were homogenized using a Branson Ultrasonics Sonifier 250 (Branson Ultrasonics Corporation, Danbury, CT, USA) in the lysis buffer, composed of 4% sodium dodecyl sulfate (SDS) in 50-mM triethylammonium bicarbonate (TEAB). The homogenized samples were centrifuged at 17,000*g* at 4°C for 5 minutes to collect pellets. The supernatant was transferred to a new tube and stored at ‒80°C for later use. The pellets in 50 µL of the lysis buffer were subjected to 60 pressure cycles, consisting of 40,000 psi for 50 seconds followed by ambient pressure for 10 seconds at 90°C, using a Barocycler 2320EXT (Pressure Biosciences, Inc., Medford, MA, USA). After the barocycling, the protein lysates were combined with the supernatant saved in the previous step, and protein concentration was estimated using a bicinchoninic acid assay. Then, 100 µg proteins for each sample were reduced and alkylated with 10-mM tris (2-carboxyethyl) phosphine hydrochloride and 40-mM chloroacetamide (CAA) at room temperature for 1 hour.

To remove SDS in the samples before enzyme digestion, the proteins in the samples were precipitated using the methanol–chloroform precipitation method followed by reconstituting in 8-M urea in 50-mM TEAB. The protein samples were digested with LysC (Wako Pure Chemical Industries, Ltd., Osaka, Japan) at a ratio of 1:100 at 37°C for 3 hours. After the urea was diluted to a concentration of 2 M by adding three volumes of 50-mM TEAB, the samples were further digested with sequencing-grade trypsin (Promega, Madison, WI, USA) at a ratio of 1:50 at 37°C overnight. The digested peptide samples were desalted with strong cation-exchange Thermo Scientific StageTips and then labeled with 10-plex TMT reagents according to the manufacturer's instructions (Thermo Fisher Scientific, Waltham, MA, USA). The eight channels (126, 127N, 127C, 128N, 128C, 129N, 129C, and 130N) were used for the labeling. The labeling reaction was performed for 1 hour at room temperature, followed by quenching with 1 volume of 100-mM Tris-HCl (pH 8.0).

The Agilent 1260 Infinity Capillary LC System (Agilent Technologies, Santa Clara, CA, USA) was used for basic reverse-phase liquid chromatographic fractionation and includes a binary pump, variable wavelength detector, an autosampler, and an automatic fraction collector. Dried samples were reconstituted in solvent A (10-mM TEAB, pH 8.5) and loaded onto an Agilent Zorbax 300Extend-C18 HPLC column (4.6 mm × 25 cm, 5 µm; Agilent Technologies). Peptides were resolved using a gradient of 2% to 50% solvent B (10-mM TEAB in 90% acetonitrile, pH 8.5) at a flow rate of 0.3 mL/min over 100 minutes, collecting 96 fractions. Subsequently, the fractions were concatenated into 24 fractions followed by vacuum drying using a SpeedVac vacuum concentrator (Thermo Fisher Scientific). The dried peptides were suspended in 30 µL of 0.1% formic acid, and 15 µL was injected.

The fractionated peptides were analyzed on an Orbitrap Fusion Lumos Tribrid Mass Spectrometer coupled with an Ultimate 3000 RSLCnano nano-flow liquid chromatography system (Thermo Fisher Scientific). The peptides from each fraction were loaded on an Acclaim PepMap100 Nano-Trap Column (100 µm  ×  2 cm; Thermo Fisher Scientific) packed with 5-µm C_18_ particles at a flow rate of 8 µL/min. Peptides were resolved at a 300-nL/min flow rate using a linear gradient of 8% to 35% solvent B (0.1% formic acid in 95% acetonitrile) over 95 minutes on an EASY-Spray HPLC column (75 µm × 50 cm; Thermo Fisher Scientific) packed with 2-µm C_18_ particles and fitted with an EASY-Spray ion source that was operated at a voltage of 2.0 kV.

Mass spectrometry analysis was completed in a data-dependent manner with a full scan in the mass-to-charge ratio (*m*/*z*) range of 300 to 1800 at the top speed setting, 3 seconds per cycle. MS1 was acquired for the precursor ions measured at a resolution of 120,000 at an *m*/*z* of 200. An MS2 scan was acquired by fragmenting precursor ions using a higher-energy collisional dissociation (HCD) method and detected at a mass resolution of 50,000 at an *m*/*z* of 200. Automatic gain control for MS1 was set to 1 million ions, and MS2 was set to 0.05 million ions. Maximum ion injection times were set to 50 milliseconds (ms) for MS1 and 100 ms for MS2 (HCD was set to 35%). The precursor isolation window was set to 1.6 *m*/*z* with a 0.4 *m*/*z* offset. Dynamic exclusion was set to 30 seconds, and singly charged ions were rejected. Internal calibration was carried out using the lock mass option (*m/z* 445.1200025) from ambient air.

Proteome Discoverer 2.2 (Thermo Fisher Scientific) was used for quantitation and identification. During MS/MS preprocessing, the top 10 peaks in each window of 100 *m*/*z* were selected for database search. The MS/MS data were then searched using SEQUEST algorithms against a mouse UniProt database (released in May 2018) with common contaminant proteins. The search parameters were as follows: (1) trypsin as a proteolytic enzyme (with up to two missed cleavages); (2) peptide mass error tolerance of 10 ppm; (3) fragment mass error tolerance of 0.02 Da; and (4) carbamidomethylation of cysteine (+57.02146 Da) and TMT tags (+229.162932 Da) on lysine residues and peptide N-termini as a fixed modification and oxidation of methionine (+15.99492 Da) as a variable modification. The minimum peptide length was set to seven amino acids, and proteins identified by only one peptide were filtered out. Peptides and proteins were filtered at a 1% false-discovery rate (FDR) at the peptide spectrum match (PSM) level using a percolator node and at the protein level using the protein FDR validator node, respectively.

The protein quantification was performed using the following parameters and methods. The most confident centroid option used for the integration mode, and the reporter ion tolerance was set to 20 ppm. MS order was set to MS2. Both unique and razor peptides were used for peptide quantification. Protein groups were considered for peptide uniqueness. Missing intensity values were replaced with the minimum value. Reporter ion abundance was computed based on the signal-to-noise ratio. Quantification value corrections for isobaric tags were disabled. The co-isolation threshold was set to 50%. The average reporter signal-to-noise threshold was set to 50. Data normalization was disabled. Protein grouping was performed by applying strict parsimony principle as follows: (1) all proteins sharing the same set or subset of identified peptides were grouped; (2) protein groups having no unique peptides among the considered peptides were filtered out; (3) Proteome Discoverer iterated through all spectra and selected which PSMs to use in ambiguous cases to make a protein group with the highest number of unambiguous and unique peptides; and (4) final protein groups were generated.

Proteome Discoverer summed all of the reporter ion intensities of PSMs for the corresponding proteins in TMT run. Finally, the protein table exported from Proteome Discoverer was imported into Perseus 1.6.0.7 software for normalization.^24^ The reporter ion intensities of four CS-exposed replicates were divided by the reporter ion intensity value of four control replicates, separately. To remove systemic deviation, each column was divided by the median value of the corresponding column.

The abundance values of reporter ion intensities from eight-plex TMT (from the Proteome Discoverer platform) were imported into Partek Genomics Suite 6.6 (Partek, Inc., St. Louis, MO, USA) for protein annotation and differential expression analysis. The normalized reporter ion intensities were examined for the standard deviation (SD) to investigate the differential expression in CS-exposed mice compared with Ct mice. The *P* values were estimated using a two-tailed *t*-test, assuming a hypothesized mean of 0 change. The normalized ratios were converted to log2 scale (becoming the conventional log-ratios or log2 fold changes) for statistical and graphic representation.

### Gene Ontology Functional Enrichment Analysis

The differentially expressed (*P* < 0.05) CE proteins from CS-exposed mice were functionally annotated using Visual Annotation Display (VLAD) 1.6.0, a web-based tool from Mouse Genome Informatics.^25^ The VLAD performs statistical analysis to test the enrichment of gene ontology (GO) terms based on their annotations to gene function. A complete set of mouse genes was used as a reference annotation dataset, and ontological terms annotated with the evidence code ND (no biological data) were excluded from the enrichment analysis. The statistically significant enriched terms were sorted based on their corrected *P* value (≤ 0.01) calculated using multiple testing and positive FDR for each term.

Finally, differentially expressed (*P* < 0.05) CE proteins were examined using the PANTHER (Protein ANalysis THrough Evolutionary Relationships) classification system, version 15.0, to annotate classes of the proteins that were differentially expressed.^26^ Briefly, differentially expressed CE proteins were subjected to the PANTHER Over-representation Test (Released 20200728) and compared with the mouse reference gene dataset (PANTHER database) to determine the under- and the over-represented protein families. Fisher's exact test with FDR and multiple test correction (*q* < 0.05) using the Benjamini–Hochberg procedure was employed to determine statistically significant protein classes.

## Results

**Figure 1.**
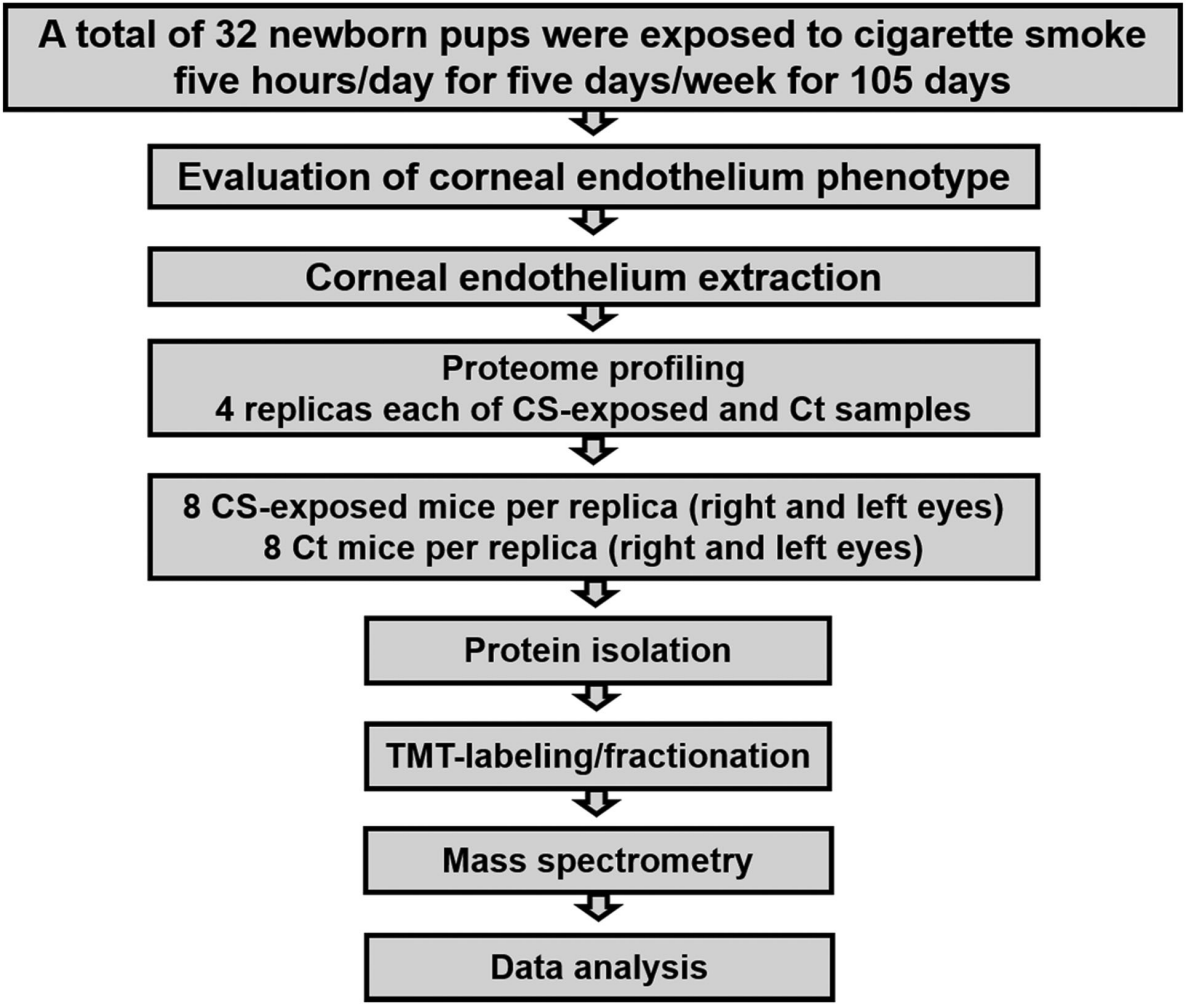
Workflow depicting the protocol used for the characterization of corneal endothelium (CE) from cigarette smoke-exposed (CS-exposed) and control (Ct) mice through a proteome profiling approach. Briefly, 32 newborn mice were exposed to CS in a smoking chamber for 5 h/d, 5 d/wk, for a total of 105 days (3.5 months). Subsequent to exposure, the CS-exposed and age-matched Ct mice were examined for CE-associated abnormalities using the ConfoScan4. The CS-exposed and Ct mice were euthanized, and the CE was peeled and used for mass spectrometry-based proteome profiling. The CS-exposed and Ct groups consisted of two male and two female biological replicates.

In this study, we evaluated molecular changes in the CE, especially DM proteins, when exposed to CS through a TMT-based proteome profiling approach (Fig. 1). A total of four 14-week-old pregnant mice (gestation days 18–20) were housed in a whole-body exposure smoking chamber, and a few days later pups were born. A total of 32 pups born within 2 to 3 days after the pregnant mice were placed in the smoke chamber were exposed to CS for 5 h/d, 5 d/wk, for a total of 3.5 months (105 days). In parallel, four adult females and 32 pups (16 males and 16 females) were housed at Johns Hopkins animal facility for 3.5 months and served as the control. After completion of the CS exposure, the CEs of the CS-exposed and the Ct mice were examined for morphologic changes and changes in CE cell density resulting from exposure to CS.

The CE images of CS-exposed mice captured by the ConfoScan4 show a difference in the shape of CECs (Supplementary Figs. S1–S36). Our analyses identified an increase (*P* = 0.005) in polymegathism (variation in size) and induction (*P* = 0.052) of pleomorphism (variation in shape) of CECs due to exposure to CS. The CE cell density in CS-exposed and Ct mice had an average of 2216.64 ± 160 cells/mm^2^ and 2421.01 ± 236 cells/mm^2^, respectively. Taken together, these data suggest nearly a 10% decrease (*P* < 0.00003) in CEC density resulting from exposure to CS (Fig. 2).

**Figure 2.**
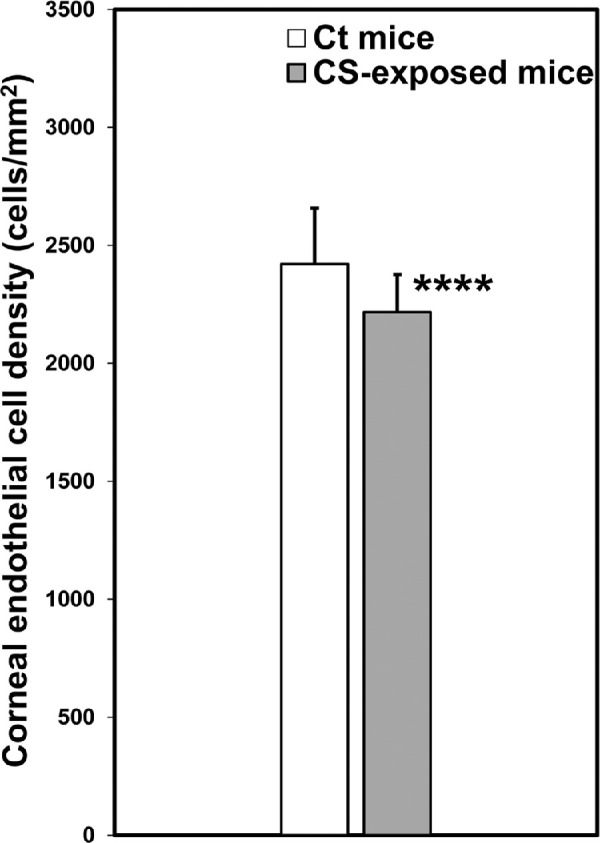
Evaluation of corneal endothelial cell (CEC) density in cigarette smoke-exposed (CS-exposed) and control (Ct) mice corneal endothelium (CE). The analysis shows a bilateral decrease in CEC density in CS-exposed mice compared with Ct mice. ^****^P < 3.0e^−05^ (two-tailed Student's *t*-test).

Next, we performed proteome profiling of CEs from CS-exposed and Ct mice. The CE was extracted under a microscope, and the extracted CE was maintained in distinct pools to serve as biological replicates. To fulfill the requirement of 100 µg of protein for mass spectrometry, each biological replicate consisted of 16 CEs (CEs from both right and left eyes) from eight mice (four male and four female). Mass spectrometry-based proteome profiling generated a total of 115,473 PSMs, yielding 28,564 total peptides corresponding to 3644 proteins in CS-exposed and Ct mice CE (Supplementary Data S1). In addition to the above-mentioned proteins, we identified 186 proteins in the CE proteome; however, these proteins were not reliably quantified (Supplementary Data S1). The MS data for the CE from CS-exposed and Ct mice have been deposited in the ProteomeXchange Consortium via the PRIDE partner repository, with dataset identifier PXD020036.

**Figure 3.**
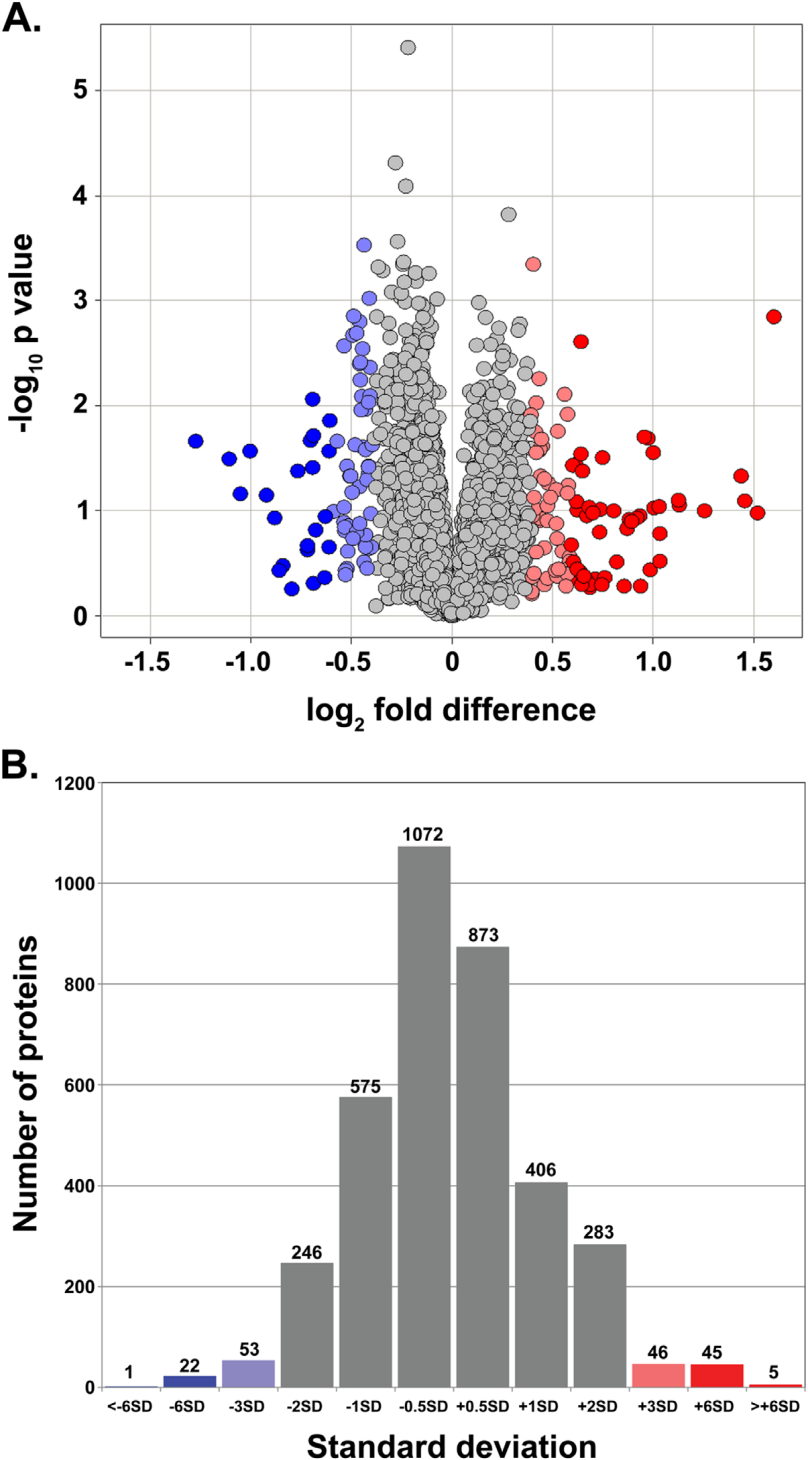
Characterization of the corneal endothelium (CE) proteome of cigarette smoke-exposed (CS-exposed) and control (Ct) mice. (**A**) Volcano plot illustration of CE proteins exhibiting changes in protein levels in CS-exposed mice compared with Ct mice. The proteome profiling identified 524 proteins (>±2 SD), including 163 exhibiting elevated levels and 361 displaying diminished levels in CS-exposed mouse CE. The fold changes are represented in the log2 scale depicted on the *x*-axis, whereas the −log10 *P* value is depicted on the *y*-axis (–log values mean that proteins with greater statistical significance are higher in the plot). Proteins that are significantly elevated are highlighted in *red* and *light red*, and those with significantly diminished levels are highlighted in *blue* and *light blue*. (**B**) Histogram illustrating the standard deviation (SD) distribution pattern of the total proteins identified in the CE proteome. The *x*-axis represents the SD, and the number of proteins is depicted on the *y*-axis. The *red* and *light red bars* represent the proteins with ≥+3 SD, and the *blue* and *light blue bars* represent the proteins with ≥–3 SD. The proteins with ±2 SDs are shown as *gray* *bars*. The number on the top of each bar represents the total number of proteins identified with a particular SD.

The proteome analysis identified a total of 524 proteins exhibiting a statistically significant change in the CE of CS-exposed mice (Fig. 3A). These included 163 and 361 proteins exhibiting elevated and reduced levels, respectively (Fig. 3A, Supplementary Data S2). Our analysis further identified 99, 67, and six proteins exhibiting ±3 SD, ±6 SD, and >±6 SD values, respectively, in the CE of CS-exposed mice (Fig. 3B).

We identified a total of 33 different collagen proteins in the CE proteome. Among these, all six type IV collagens (COL4α1, COL4α2, COL4α3, COL4α4, COL4α5, and COL4α6) exhibited diminished levels (*P* < 0.05) in CS-exposed mice CE (Table 1, Supplementary Data S2). A similar trend was observed with COL7α1, COL8α1, COL8α2, COL11α2, and COL18α1 (i.e., present in lower levels in the CE of CS-exposed mice) (Table 1, Supplementary Data S2).

**Table 1.** Subset of Proteins Exhibiting Differential Expression in the Proteome of Cigarette Smoke-Exposed (CS-Exposed) Mouse Corneal Endothelium (CE) Reported Being Part of the Descemet's Membrane (DM) of the Cornea

	Mean Value		CS-Exposed Versus Control	
Protein	Control	CS-Exposed	*P*	Fold Change
COL4α1	15.663	15.2657	0.023618	–1.31709
COL4α2	16.8888	16.4341	0.00386061	–1.37052
COL4α3	14.846	14.4785	0.000477366	–1.29006
COL4α4	15.3858	15.0516	0.00905973	–1.26075
COL4α5	15.3157	15.022	0.00361406	–1.22582
COL4α6	14.8438	14.2355	0.0137853	–1.52451
COL7α1	15.4676	15.2973	0.0411498	–1.12532
COL8α1	14.142	13.64	0.0466376	–1.41618
COL8α2	14.1729	13.7589	0.0378013	–1.33242
COL11α2	12.4389	11.9498	0.00139167	–1.40359
COL18α1	15.2934	15.1245	0.0440123	–1.1242
CLU	16.4244	16.1223	0.042934	–1.23296
FN1	12.6119	12.3586	0.00084638	–1.19198
HSPG2	19.0853	18.7088	0.0396816	–1.29817
LAMα3	17.0477	16.7778	0.000270895	–1.20571
LAMα1	14.1538	13.9357	0.00403507	–1.16321
LAMβ3	15.485	15.2821	0.00782843	–1.15101
LAMα5	17.8347	17.6246	0.0160604	–1.15672
LAMβ2	17.2045	16.9813	0.0163778	–1.16734
LAMα2	11.5651	11.2364	0.016651	–1.25588
NID1	17.4166	17.2673	0.0197851	–1.10905
NID2	16.3522	16.0855	0.0188781	–1.20305
TGFβ1	11.3954	11.2305	0.00303732	–1.1211
TGFβ2	13.1618	12.8327	0.017469	–1.25619

We identified multiple ECM proteins in the CE proteome. Among these, FN1, EGFLAM, CLU, HSPG2, VIT, NID1, NID2, and COCH exhibited diminished levels (*P* < 0.05) in CS-exposed mice CE (Table 1, Supplementary Data S2). Additionally, we identified six laminin proteins (LAMα1, LAMα2, LAMα3, LAMα5, LAMβ2, and LAMβ25) exhibiting diminished levels (*P* < 0.05) in the CE of CS-exposed mice (Table 1, Supplementary Data S2). Furthermore, we identified a total of 12 integrin proteins in the CE proteome present at lower levels in the CE of CS-exposed mice (Supplementary Data S2).

In addition to ECM proteins, we identified the expression of over 70 solute carrier proteins in the CE proteome, with the majority of them present at lower levels in the CE from CS-exposed mice (Supplementary Data S2). In particular, we identified lower levels (*P* < 0.05) of ATP11B, ATP13A1, ATP2C1, SLC39A4, SLC35B2, and SLC38A1 and higher levels (*P* < 0.05) of ATP6V1E1, SLC16A1, SLC25A31, SLC2A1, and SLC6A6 in CE from CS-exposed mice (Supplementary Data S2).

GO-based functional enrichment analysis was performed using the differentially expressed proteins identified in the CS-exposed CE proteome. The analysis revealed the enrichment of GO terms linked with ECM- and collagen-related assembly and organization (Supplementary Data S3). The analysis revealed the enrichment of biological process GO terms associated with ECM assembly (GO:0085029), ECM organization (GO:0030198 and GO:0043062), and collagen fibril organization (GO:0030199) in the CS-exposed CE proteome (Supplementary Data S3). The enrichment of six cellular component GO terms associated with ECM (GO:0031012), collagen-containing ECM (GO:0062023), basement membrane (GO:0005604), basement membrane collagen trimer (GO: 0098651), collagen network (GO: 0098645), and collagen type IV trimer (GO: 0005587) was identified in the CS-exposed CE proteome (Supplementary Data S3). Moreover, we identified the enrichment of molecular function GO terms associated with ECM binding (GO:0050840), ECM structural constituent (GO:0005201), and laminin-binding (GO:0043236) in the CS-exposed CE proteome (Supplementary Data S3).

**Figure 4.**
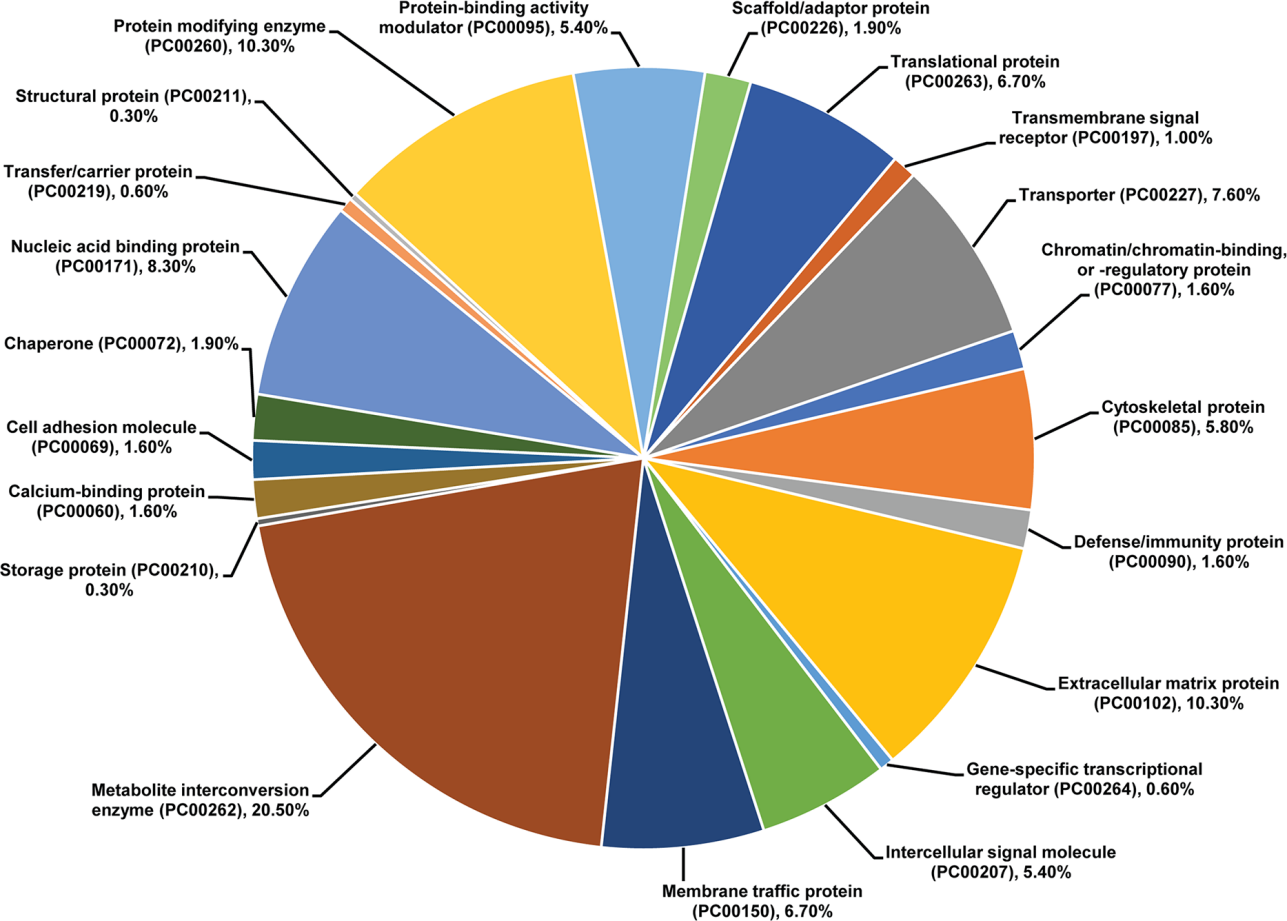
Pie chart illustrating various protein classes associated with differentially expressed corneal endothelium (CE) proteins in cigarette smoke-exposed (CS-exposed) mice. The differentially expressed CE proteins (524 proteins; *P* < 0.05) were analyzed using the PANTHER classification system to annotate the protein classes. The analysis revealed a total of 21 protein classes associated with differentially expressed CE proteins.

We further used the PANTHER tools to classify differentially expressed (*P* < 0.05) CE proteins, which identified a total of 21 protein classes (Fig. 4). Included in these proteins classes are 32 ECM (PC00102), 26 nucleic acid-binding (PC00171), 23 transporter (PC00227), and 18 cytoskeletal (PC00085) proteins (Fig. 4). Finally, we used the PANTHER tools to identify over- and under-represented protein classes among the differentially expressed (*P* < 0.05) CE proteins, which identified six protein classes (Table 2A) and six protein subclasses (Table 2B). Importantly, we identified 8.4-fold and 10.42-fold over-representation of ECM (PC00102) and ECM structural (PC00103) proteins, respectively, in differentially expressed CE proteins (Table 2), highlighting the shared volume of the ECM proteins affected by the exposure to CS.

**Table 2.** Over- and Under-Represented Protein Classes (2A) and Subclasses (2B) Identified in Differentially Expressed Corneal Endothelium (CE) Proteins

PANTHER Protein Classes	Number of Differentially Expressed Proteins Identified in CS-Exposed CE	Fold Enrichment	*P*	FDR
Table 2A				
Extracellular matrix protein (PC00102)	32	+8.4	1.92e^−18^	3.75e^−16^
Translational protein (PC00263)	21	+3.08	1.19e^−05^	2.58e^−04^
Metabolite interconversion enzyme (PC00262)	64	+1.89	1.74e^−06^	4.86e^−05^
Membrane traffic protein (PC00150)	21	+2.29	6.55e^−04^	9.12e^−03^
Transmembrane signal receptor (PC00197)	3	–0.18	7.21e^−05^	1.28e^−03^
Gene-specific transcriptional regulator (PC00264)	2	–0.08	6.45e^−09^	2.52e^−07^
Table 2B				
Transferase (PC00220)	26	+2.23	2.73e^−04^	4.09e^−03^
Extracellular matrix structural protein (PC00103)	18	+10.42	2.66e^−12^	2.59e^−10^
Actin or actin-binding cytoskeletal protein (PC00041)	14	+2.51	2.16e^−03^	2.64e^−02^
Dehydrogenase (PC00092)	9	+3.2	2.96e^−03^	3.40e^−02^
Aminoacyl-tRNA synthetase (PC00047)	5	+6.21	1.95e^−03^	2.54e^−02^
DNA-binding transcription factor (PC00218)	2	–0.09	4.39e^−08^	1.43e^−06^

The expected number of proteins in the mouse reference database was compared with the observed number of proteins differentially expressed in CS-exposed CE, resulting in enrichment (over- or underrepresentation as designated by the + and – signs, respectively) of protein classes.

## Discussion

Here, we report changes at a molecular level in the CE of mice exposed to chronic CS. Our data confirm that exposure to CS results in reduced CEC density accompanied by diminished levels of multiple collagen and ECM proteins associated with DM. To the best of our knowledge, this is the first report investigating the effects of CS on the CE through MS-based proteome profiling in mice.

CS is a major risk factor contributing to multiple ophthalmological disorders.^27^^–^^29^ To date, only a few clinical studies investigating the effects of CS on the CE have been performed.^20^^,^^30^^,^^31^ Kara and colleagues^30^ performed specular microscopy-based corneal evaluation in a small study cohort (25 chronic smokers and 21 age-matched non-smokers) and identified reduced CEC density (not significant) in smokers. Ilhan and colleagues^20^ performed non-contact specular microscopy to investigate CCT and CEC density in 103 cigarette smokers and 106 non-smokers and identified a significant decrease in CEC density in smokers. In another study, Karakurt and colleagues^31^ profiled smokers based on cigarette consumption (packs per year) and reported a significant decrease in CEC density in chronic smokers who smoked ≥20 packs per year. Zhang and colleagues^9^ reported a significant association of CS with advanced Fuchs endothelial corneal dystrophy (FECD) development in a large study group. Moreover, increased susceptibility of oxidative stress-induced apoptosis in FECD endothelial cells has been reported by multiple studies.^15^^,^^16^ These published datasets are consistent with our finding of a decrease in CEC density in mice exposed to CS.

Several investigations have shown the susceptibility of CE to oxidative stress.^12^^–^^16^ Shin and colleagues^14^ reported that CLU (clusterin), a glycoprotein expressed in the human CE, protects human CECs from oxidative injury-mediated cell death by inhibiting ROS production. Multiple other studies have also reported changes in the expression of CLU in patients with corneal endothelial dystrophies, including FECD and bullous keratopathy.^32^^,^^33^ The downregulation of CLU in CS-exposed CE indicates ROS-mediated oxidative stress, which is further evident by low levels of SOD3 in the CS-exposed CE. SOD3 is a major superoxide dismutase enzyme in the human cornea, and it has been reported that lack of SOD3 results in elevated levels of ROS and age-related loss of CECs in SOD3-null mice.^12^^,^^13^

DM in the cornea is composed of multiple collagen proteins, including type IV and type VIII.^34^ The type IV collagens (α1–α6) are integral components of DM, and disruption of these collagens results in multiple corneal abnormalities.^35^^,^^36^ Mutations of COL4α1 result in DM defects that lead to anterior segment dysgenesis and corneal opacifications in humans and mice.^35^^–^^37^ Recently, Ronnow and colleagues^38^ investigated COL4α3 degradation in the alveolar basement membrane in chronic obstructive pulmonary disease patients and identified reduced levels of COL4α3, consistent with lower levels of COL4α3 identified in CS-exposed CE in our study.

The precise mechanism of the events that result in diminished levels of ECM proteins in CS-exposed CE remains unanswered. We identified low levels of TGFβ1 and TGFβ2 proteins in CS-exposed CE and, because both TGFβ1 and TGFβ2 proteins mediate expression of collagens, fibronectin, and integrins, it is possible that the diminished expression of TGFβ1 and TGFβ2 may have contributed to the decrease in ECM proteins. However, because DM is secreted by the CECs, it is equally possible that the loss of endothelial cells following CS treatment could be responsible for the lower levels of collagens, integrins, and glycoproteins associated with DM. Finally, we cannot rule out the possibility that both of the above-mentioned scenarios contribute to the diminished levels of ECM proteins in CS-exposed CE.

In conclusion, we report changes at a molecular level in the CE of mice exposed to chronic CS, and our data confirm that exposure to CS results in reduced CEC density accompanied by diminished levels of multiple collagen and ECM proteins associated with DM. The results reported in this study will add to our understanding of CE dystrophies resulting from exposure to CS and help in the development of therapeutic strategies to combat CS-exposure-related loss of vision.

## Supplementary Material

Supplement 1

Supplement 2

Supplement 3

Supplement 4
