# Supplementary material for: Cigarette Smoke Triggers Loss of Corneal Endothelial Cells and Disruption of Descemet's Membrane Proteins in Mice
Source: Invest Ophthalmol Vis Sci. 2021 Mar 2;62(3):3. doi: 10.1167/iovs.62.3.3 (PMC7938020; doi:10.1167/iovs.62.3.3)
Supplement: Supplement 4 [file iovs-62-3-3_s004.pdf]

Cigarette Smoke Triggers Loss of Corneal Endothelial Cells and Disruption of Descemet's Membrane Proteins in Mice

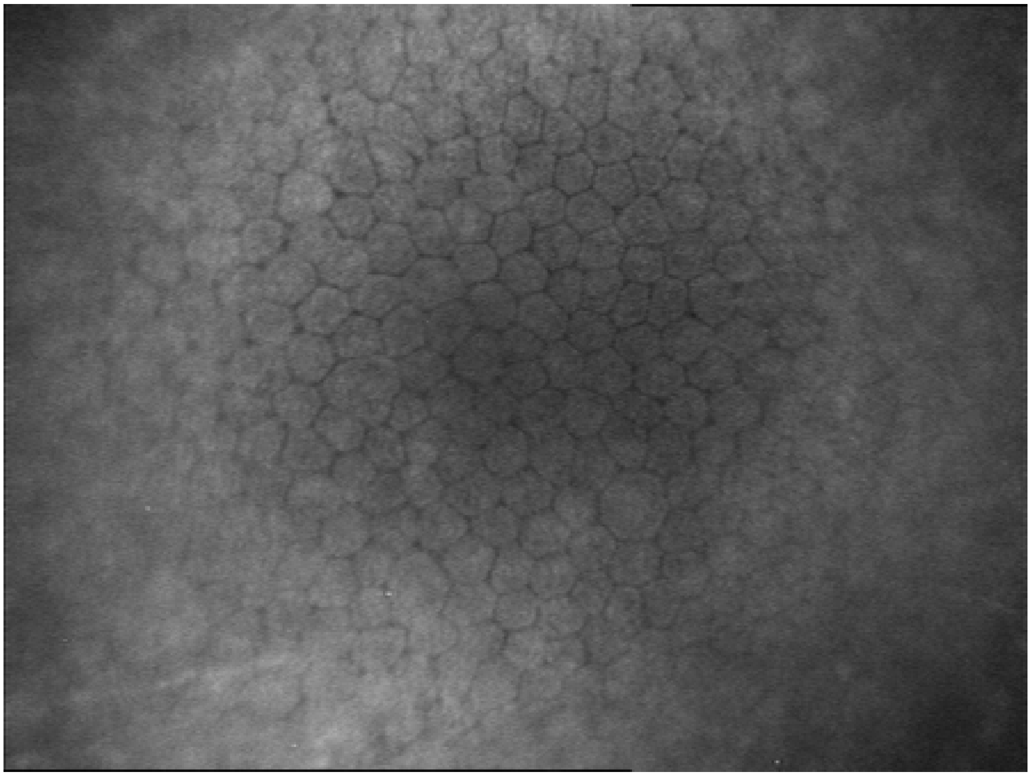

Supplementary Figure 1: Evaluation of the corneal endothelium phenotype by Confoscan4 scanning microscope in cigarette smoke-exposed mouse 1 (CS-1).

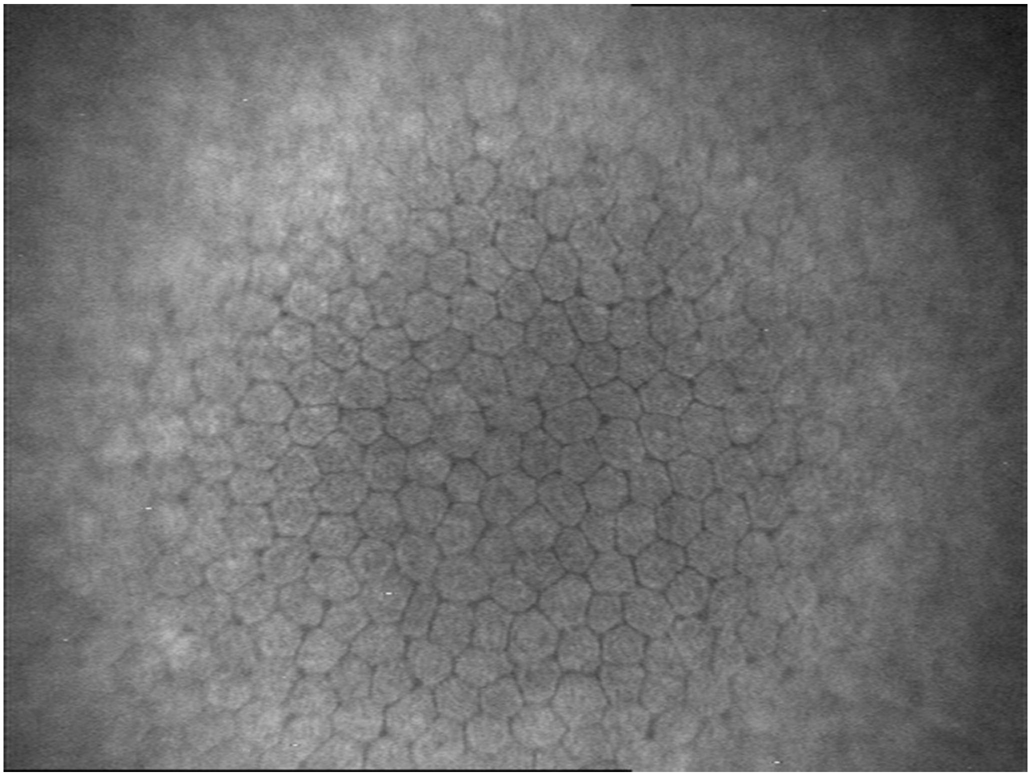

Supplementary Figure 2: Evaluation of the corneal endothelium phenotype by Confoscan4 scanning microscope in cigarette smoke-exposed mouse 2 (CS-2).

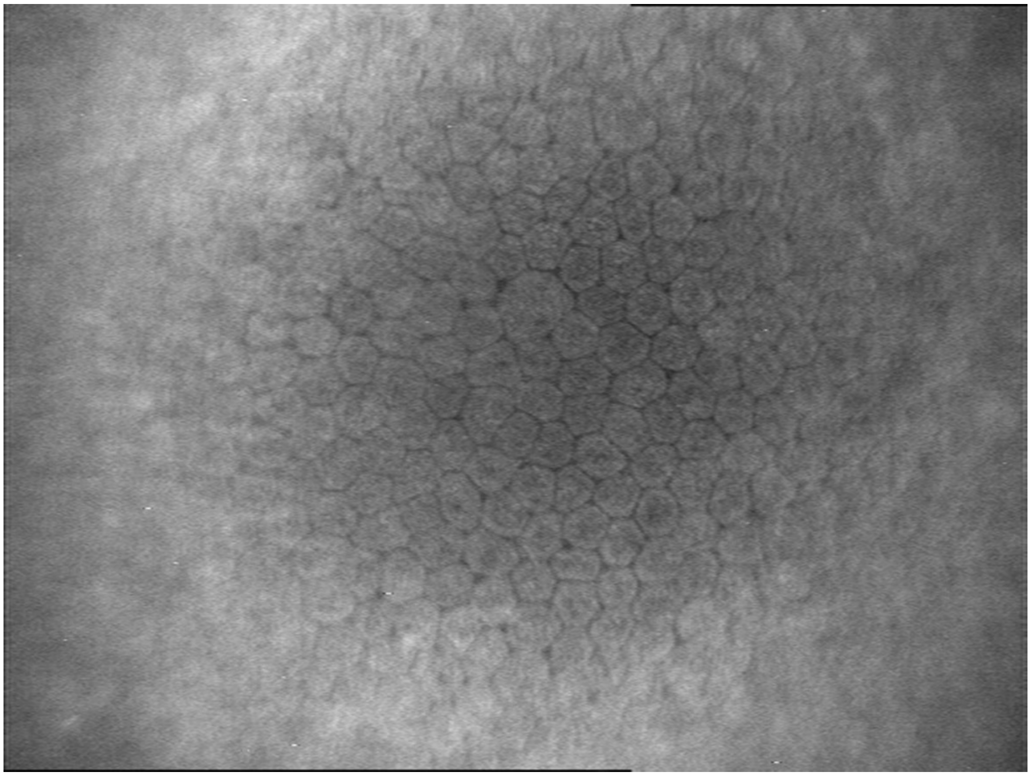

Supplementary Figure 3: Evaluation of the corneal endothelium phenotype by Confoscan4 scanning microscope in cigarette smoke-exposed mouse 3 (CS-3).

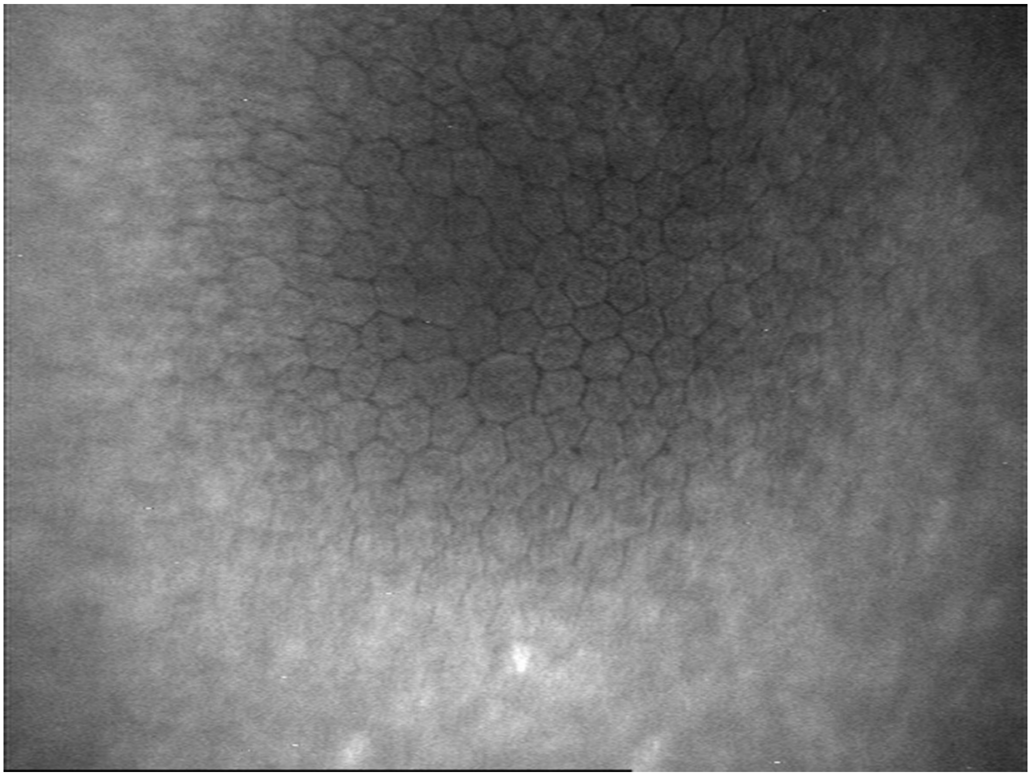

Supplementary Figure 4: Evaluation of the corneal endothelium phenotype by Confoscan4 scanning microscope in cigarette smoke-exposed mouse 4 (CS-4).

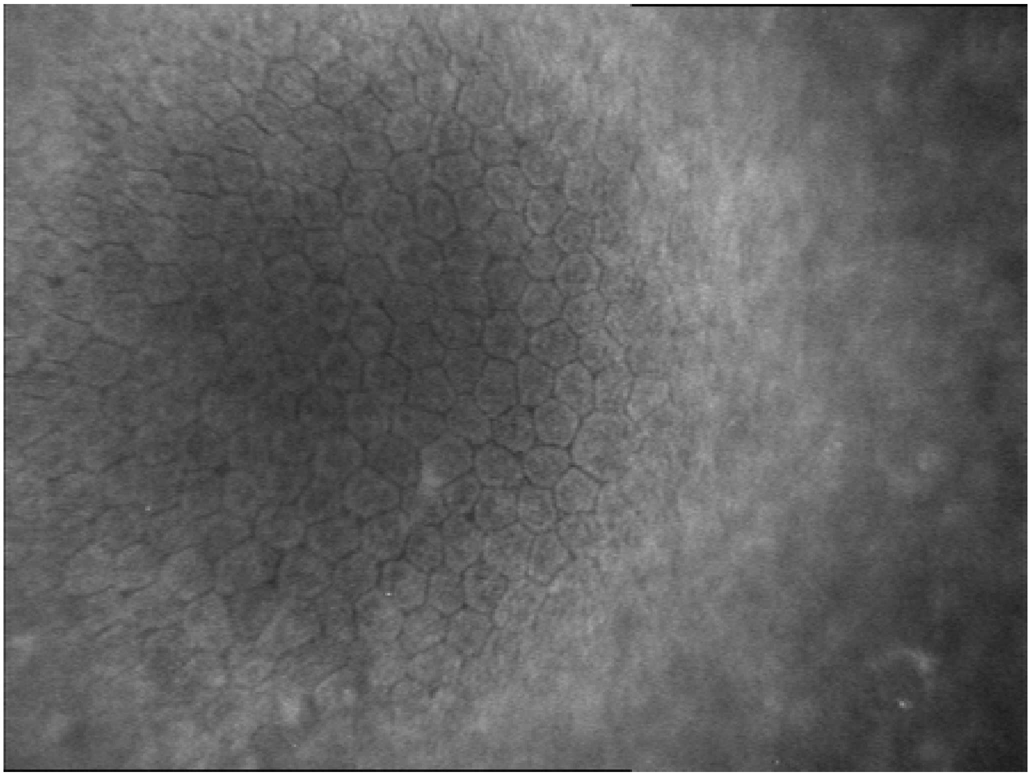

Supplementary Figure 5: Evaluation of the corneal endothelium phenotype by Confoscan4 scanning microscope in cigarette smoke-exposed mouse 5 (CS-5).

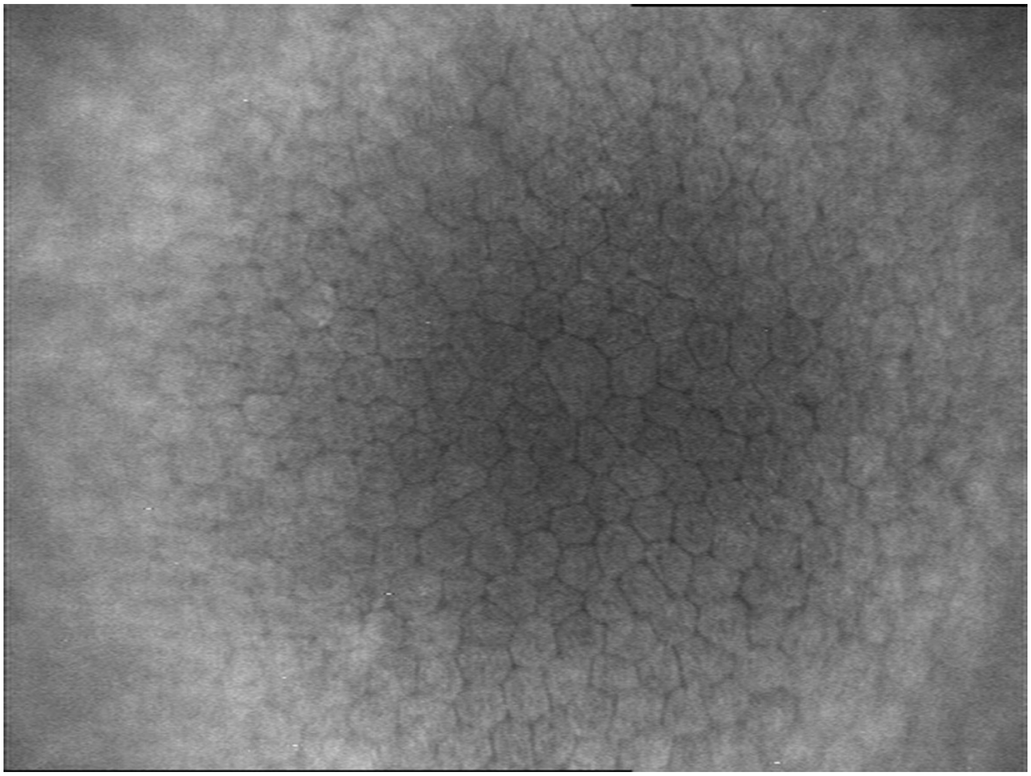

Supplementary Figure 6: Evaluation of the corneal endothelium phenotype by Confoscan4 scanning microscope in cigarette smoke-exposed mouse 6 (CS-6).

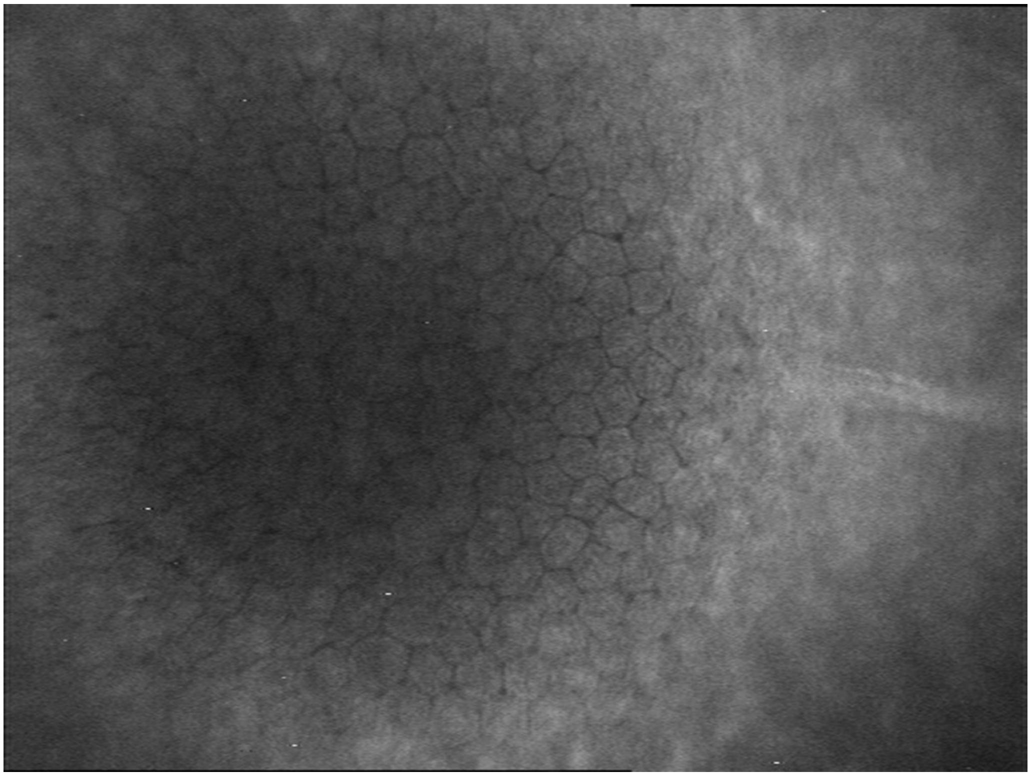

Supplementary Figure 7: Evaluation of the corneal endothelium phenotype by Confoscan4 scanning microscope in cigarette smoke-exposed mouse 7 (CS-7).

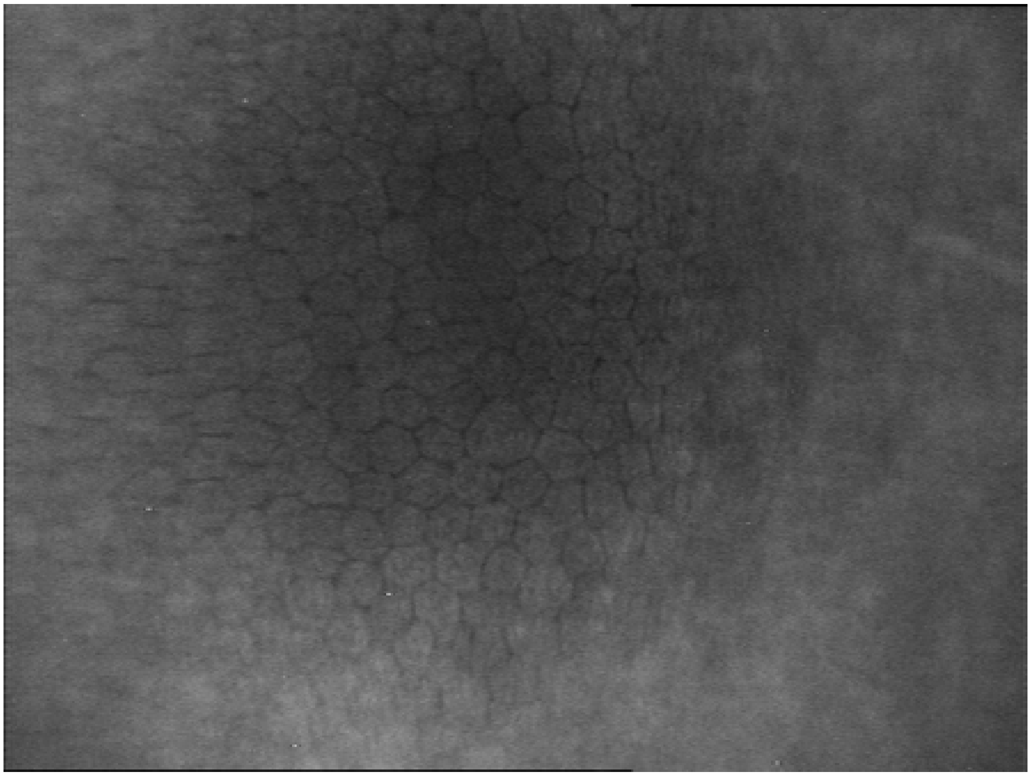

Supplementary Figure 8: Evaluation of the corneal endothelium phenotype by Confoscan4 scanning microscope in cigarette smoke-exposed mouse 8 (CS-8).

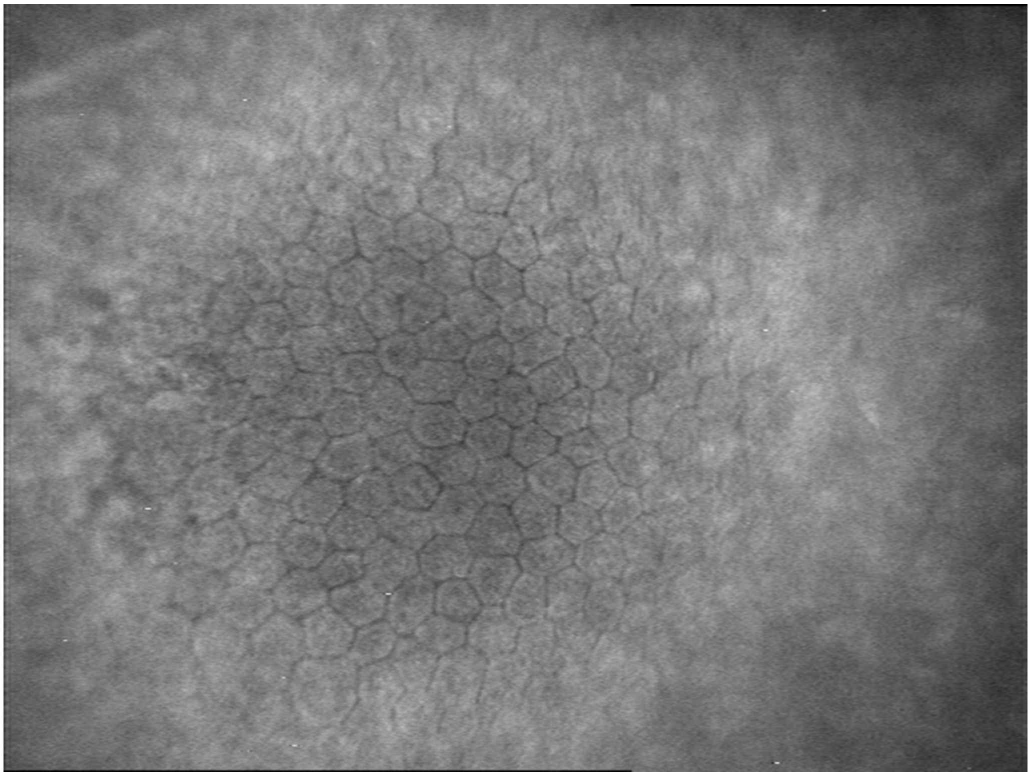

Supplementary Figure 9: Evaluation of the corneal endothelium phenotype by Confoscan4 scanning microscope in cigarette smoke-exposed mouse 9 (CS-9).

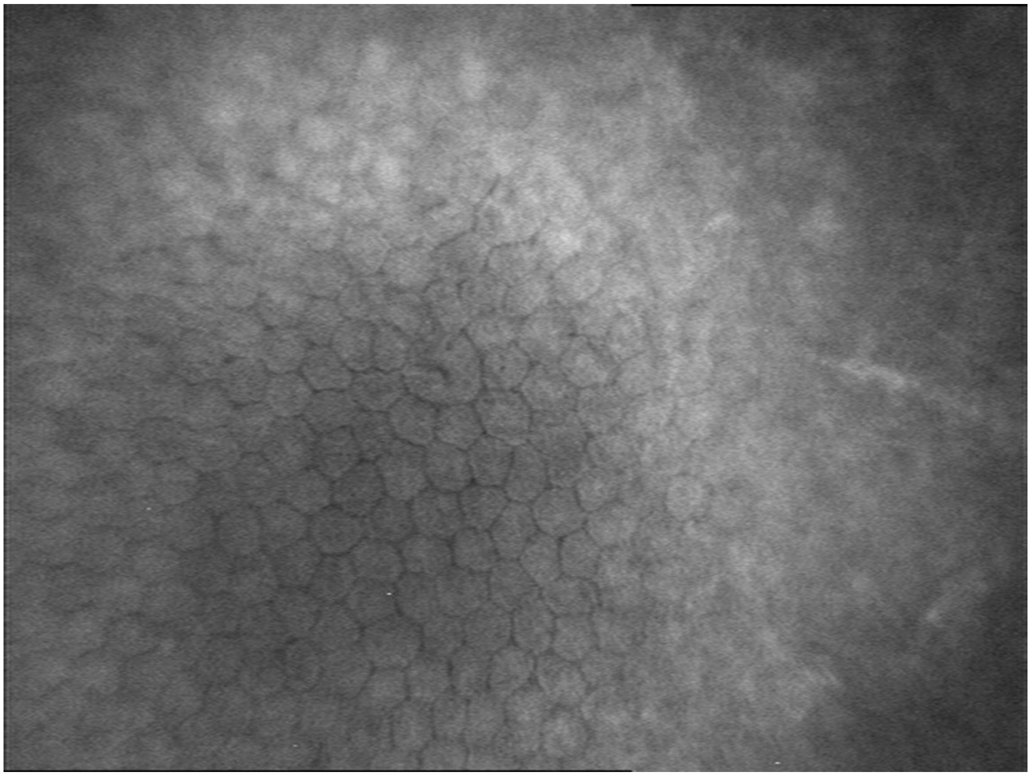

Supplementary Figure 10: Evaluation of the corneal endothelium phenotype by Confoscan4 scanning microscope in cigarette smoke-exposed mouse 10 (CS-10).

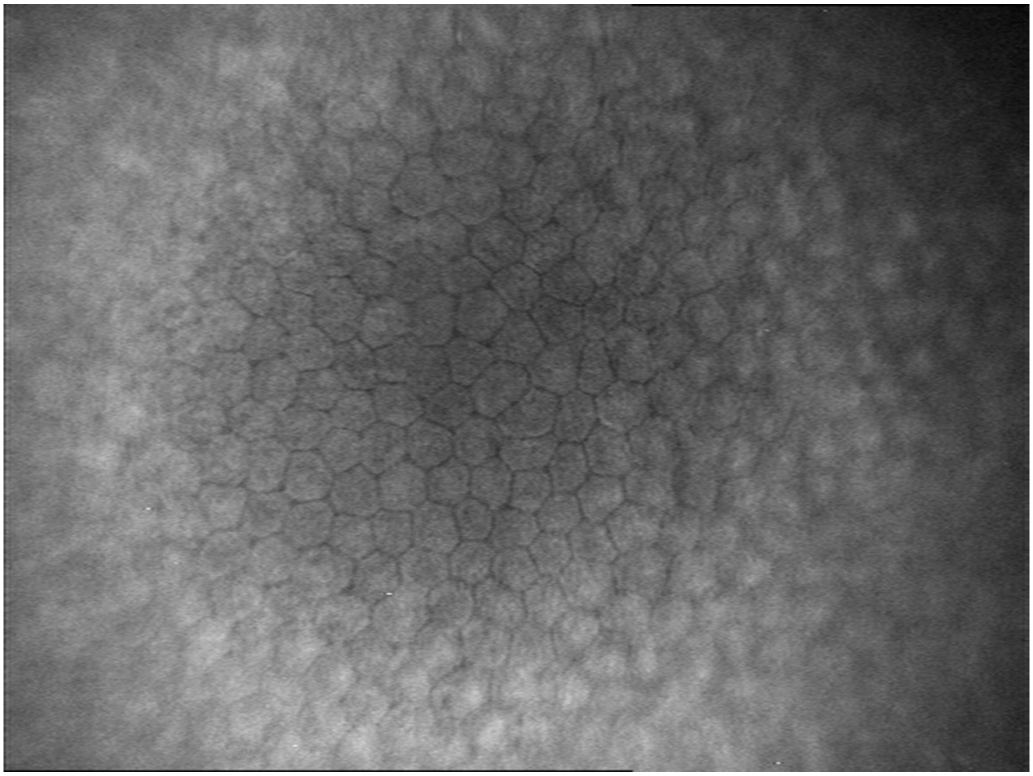

Supplementary Figure 11: Evaluation of the corneal endothelium phenotype by Confoscan4 scanning microscope in cigarette smoke-exposed mouse 11 (CS-11).

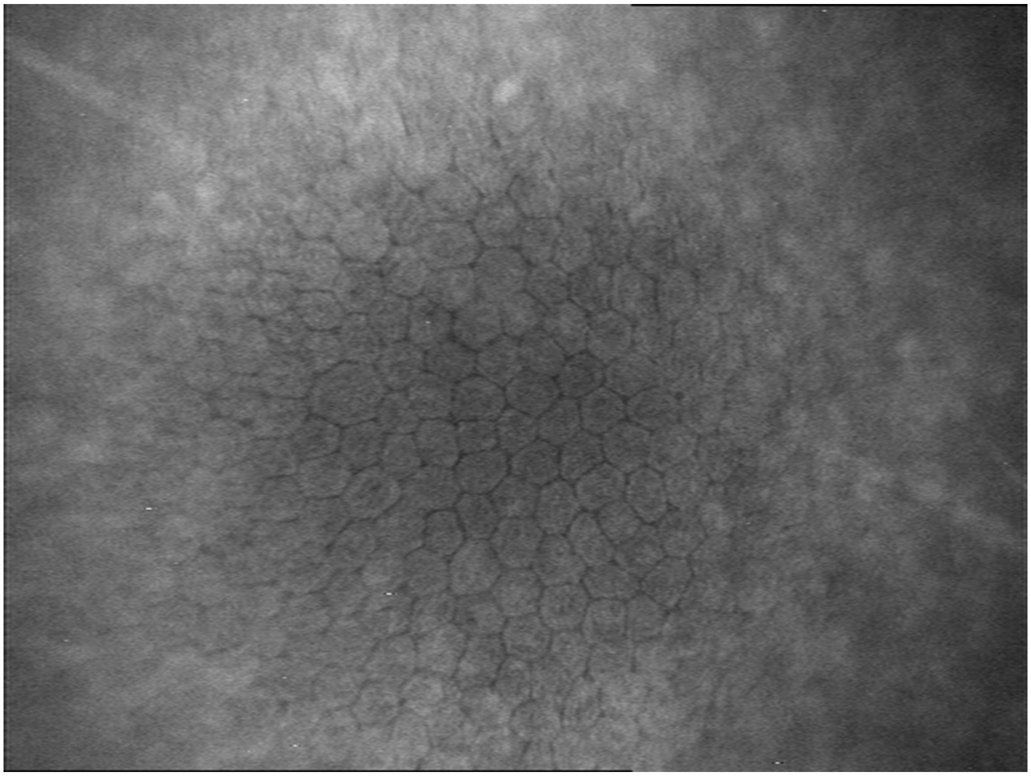

Supplementary Figure 12: Evaluation of the corneal endothelium phenotype by Confoscan4 scanning microscope in cigarette smoke-exposed mouse 12 (CS-12).

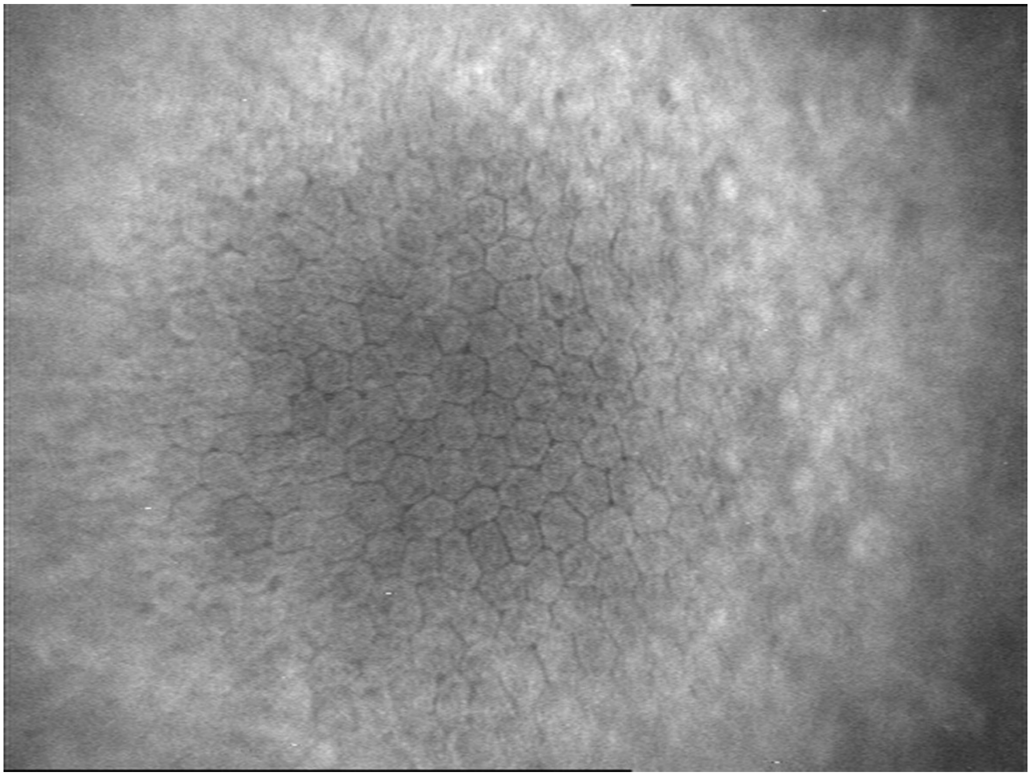

Supplementary Figure 13: Evaluation of the corneal endothelium phenotype by Confoscan4 scanning microscope in cigarette smoke-exposed mouse 13 (CS-13).

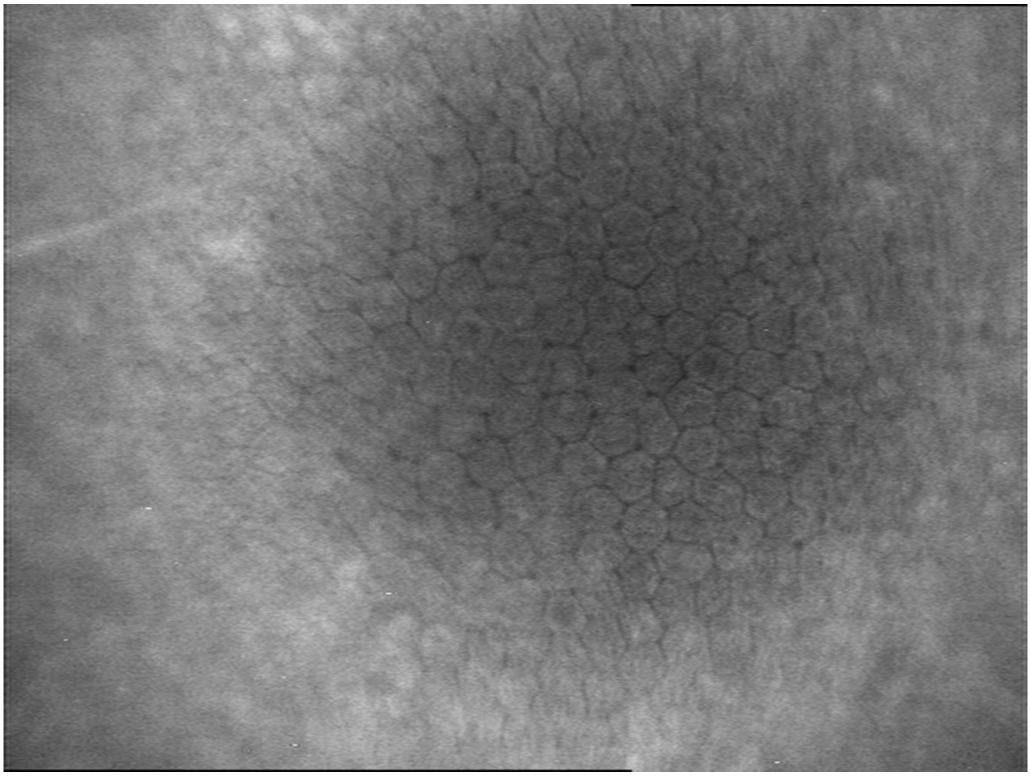

Supplementary Figure 14: Evaluation of the corneal endothelium phenotype by Confoscan4 scanning microscope in cigarette smoke-exposed mouse 14 (CS-14).

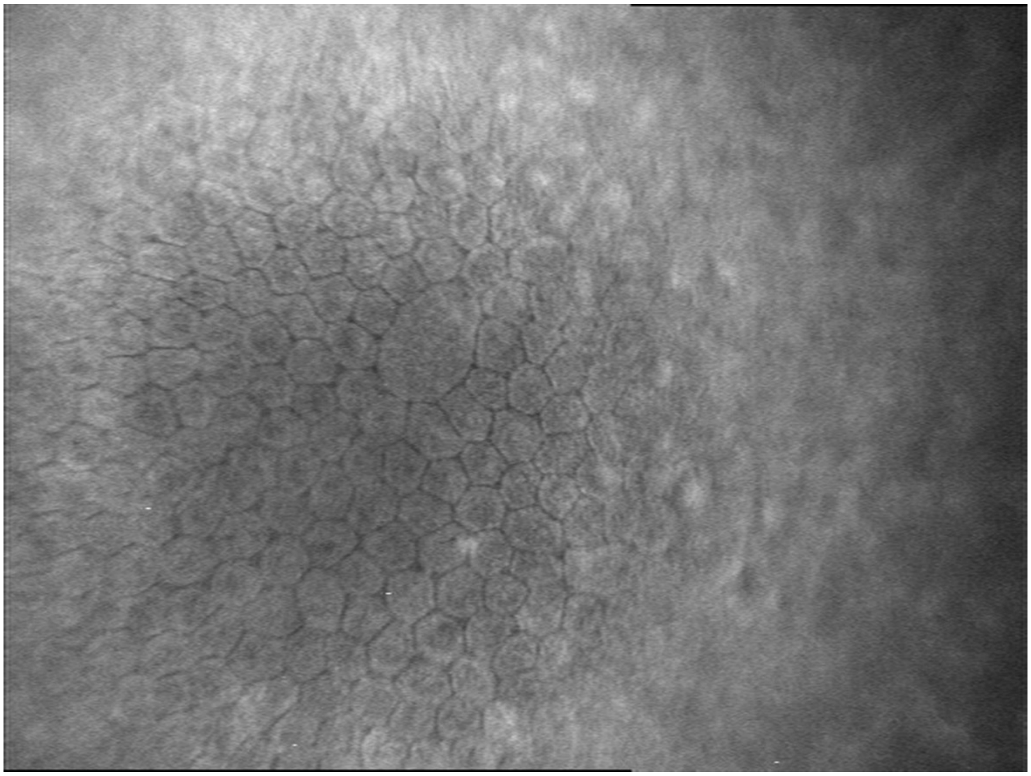

Supplementary Figure 15: Evaluation of the corneal endothelium phenotype by Confoscan4 scanning microscope in cigarette smoke-exposed mouse 15 (CS-15).

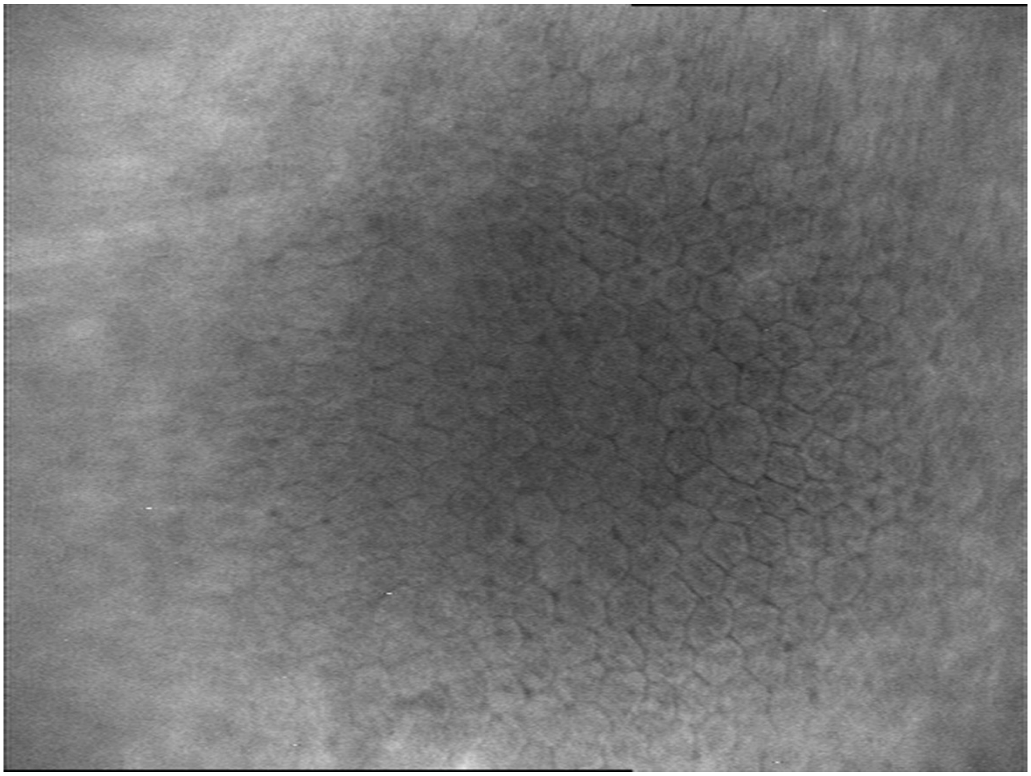

Supplementary Figure 16: Evaluation of the corneal endothelium phenotype by Confoscan4 scanning microscope in cigarette smoke-exposed mouse 16 (CS-16).

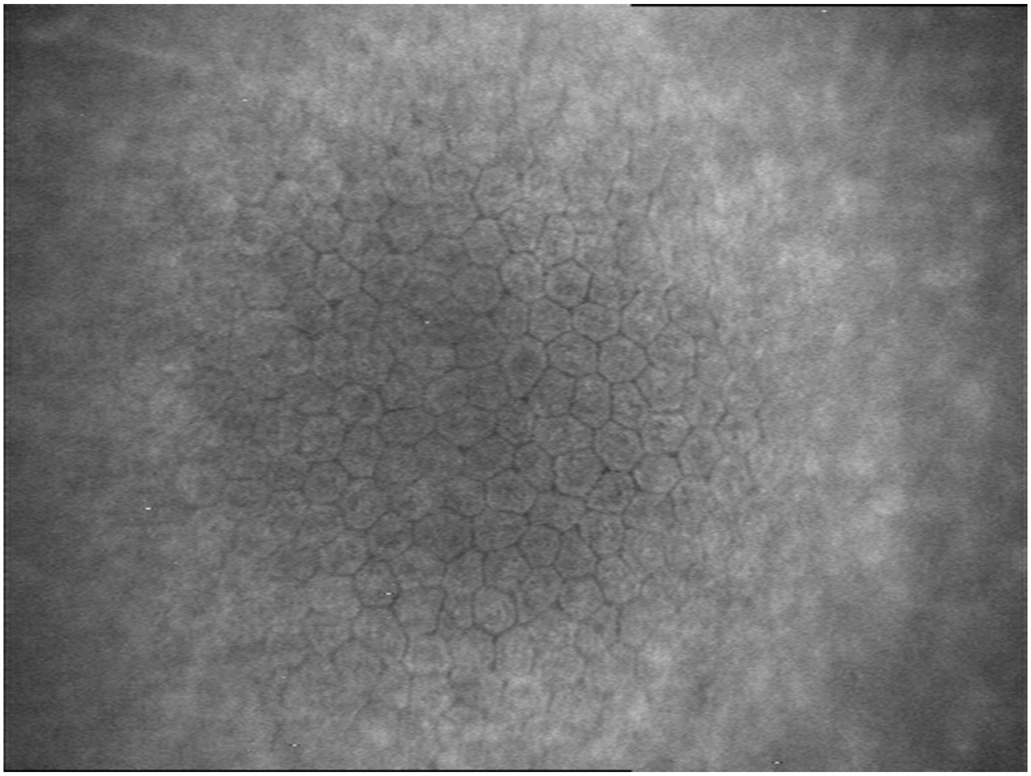

Supplementary Figure 17: Evaluation of the corneal endothelium phenotype by Confoscan4 scanning microscope in cigarette smoke-exposed mouse 17 (CS-17).

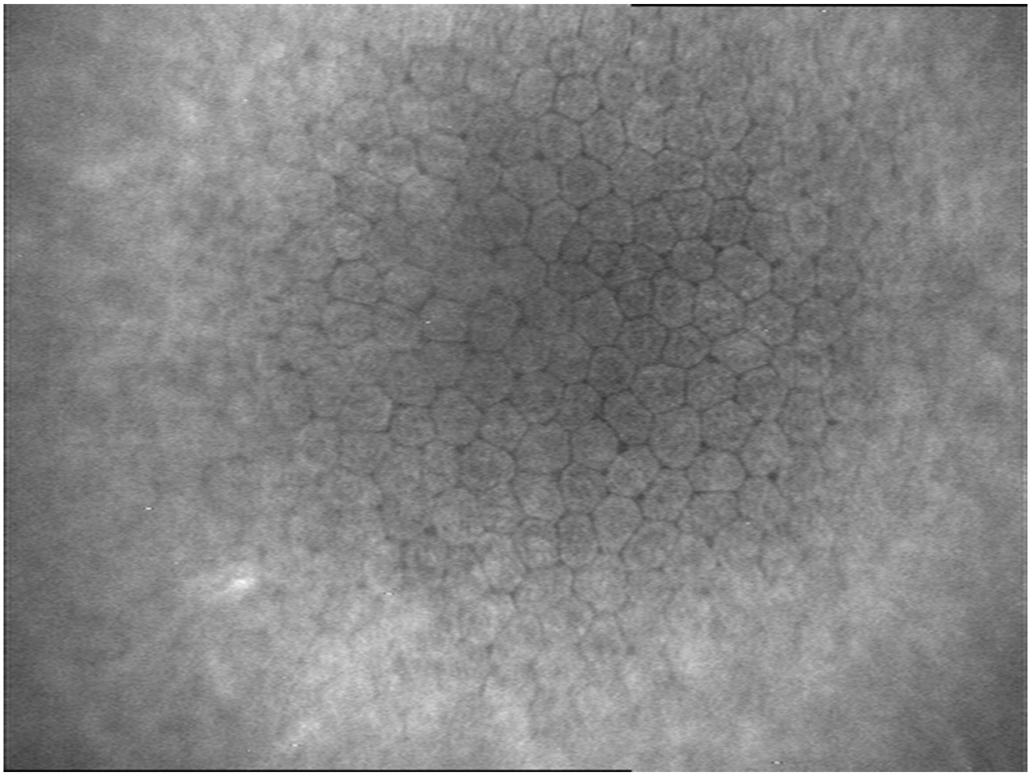

Supplementary Figure 18: Evaluation of the corneal endothelium phenotype by Confoscan4 scanning microscope in cigarette smoke-exposed mouse 18 (CS-18).

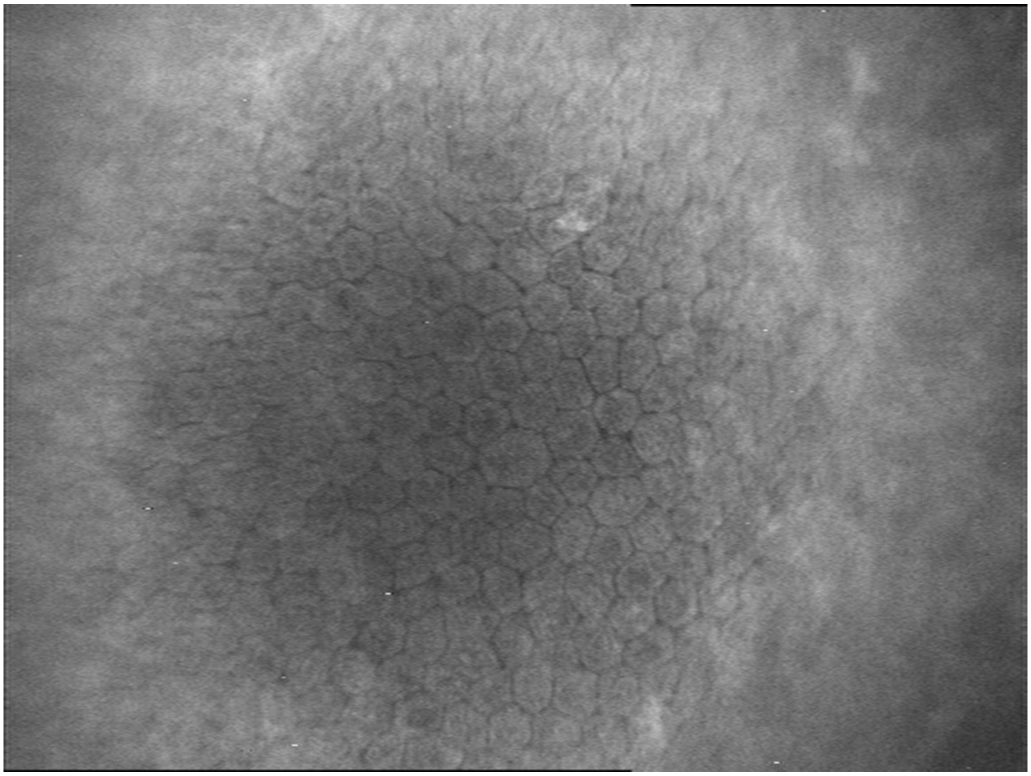

Supplementary Figure 19: Evaluation of the corneal endothelium phenotype by Confoscan4 scanning microscope in cigarette smoke-exposed mouse 19 (CS-19).

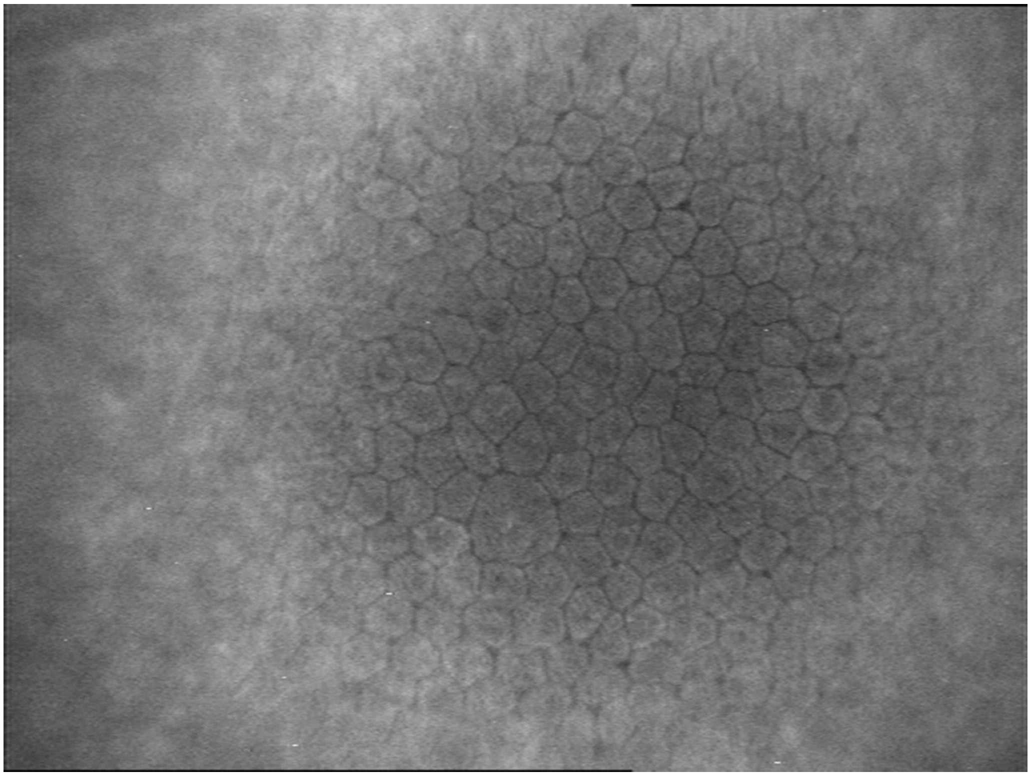

Supplementary Figure 20: Evaluation of the corneal endothelium phenotype by Confoscan4 scanning microscope in cigarette smoke-exposed mouse 20 (CS-20).

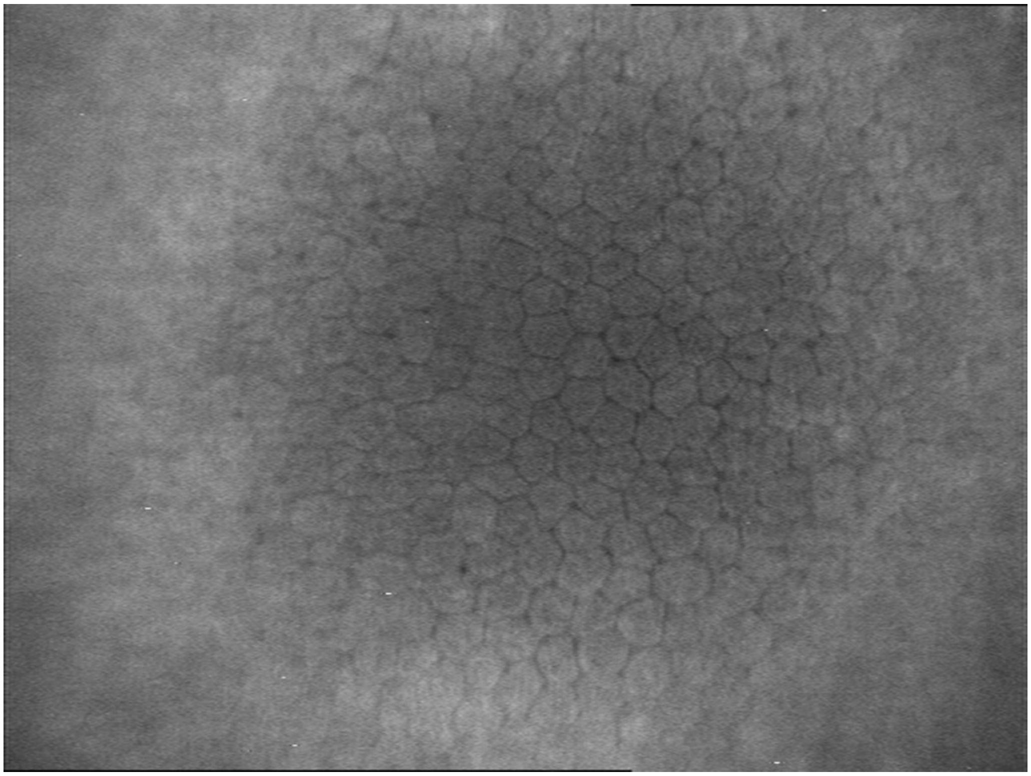

Supplementary Figure 21: Evaluation of the corneal endothelium phenotype by Confoscan4 scanning microscope in cigarette smoke-exposed mouse 21 (CS-21).

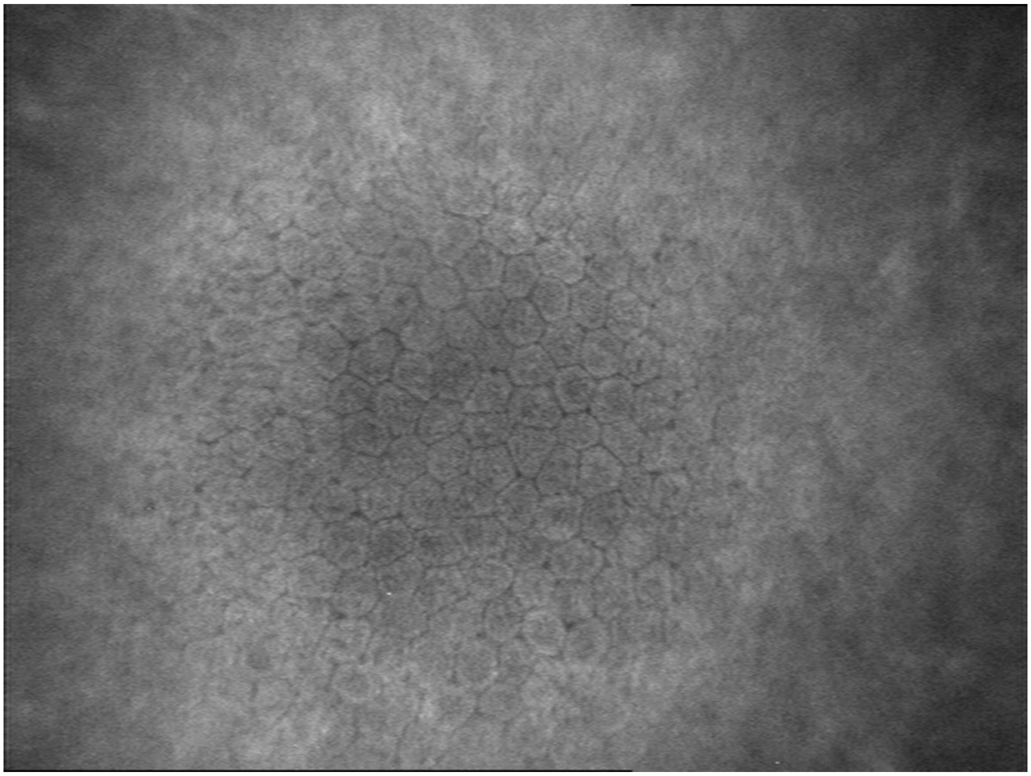

Supplementary Figure 22: Evaluation of the corneal endothelium phenotype by Confoscan4 scanning microscope in cigarette smoke-exposed mouse 22 (CS-22).

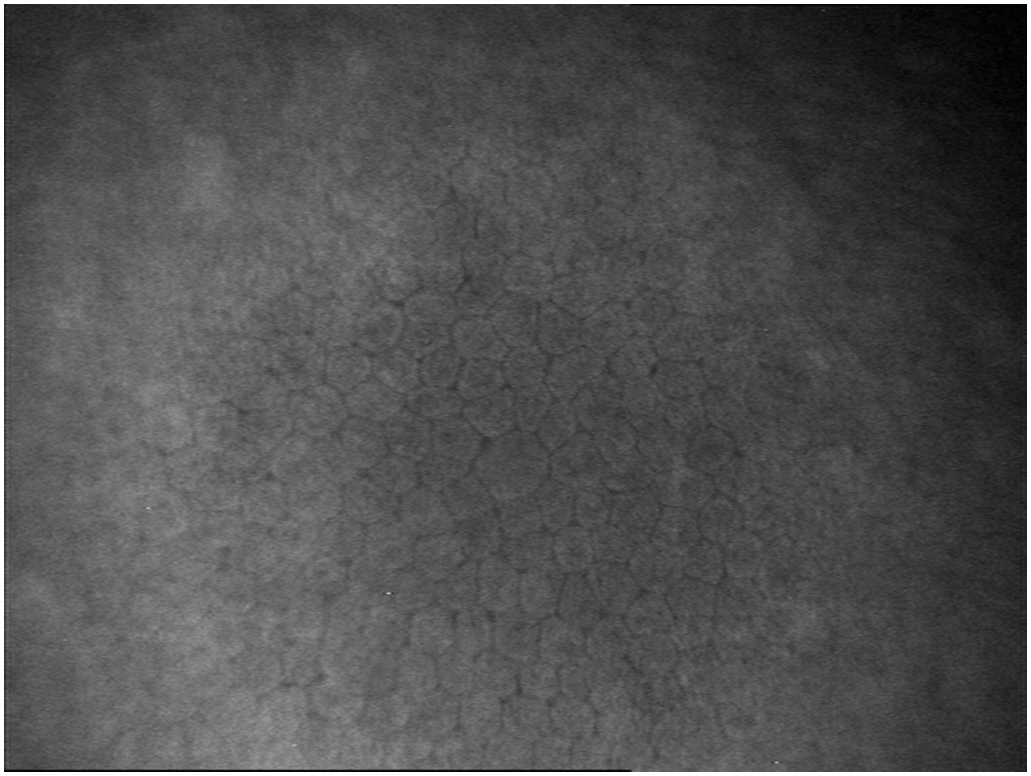

Supplementary Figure 23: Evaluation of the corneal endothelium phenotype by Confoscan4 scanning microscope in cigarette smoke-exposed mouse 23 (CS-23).

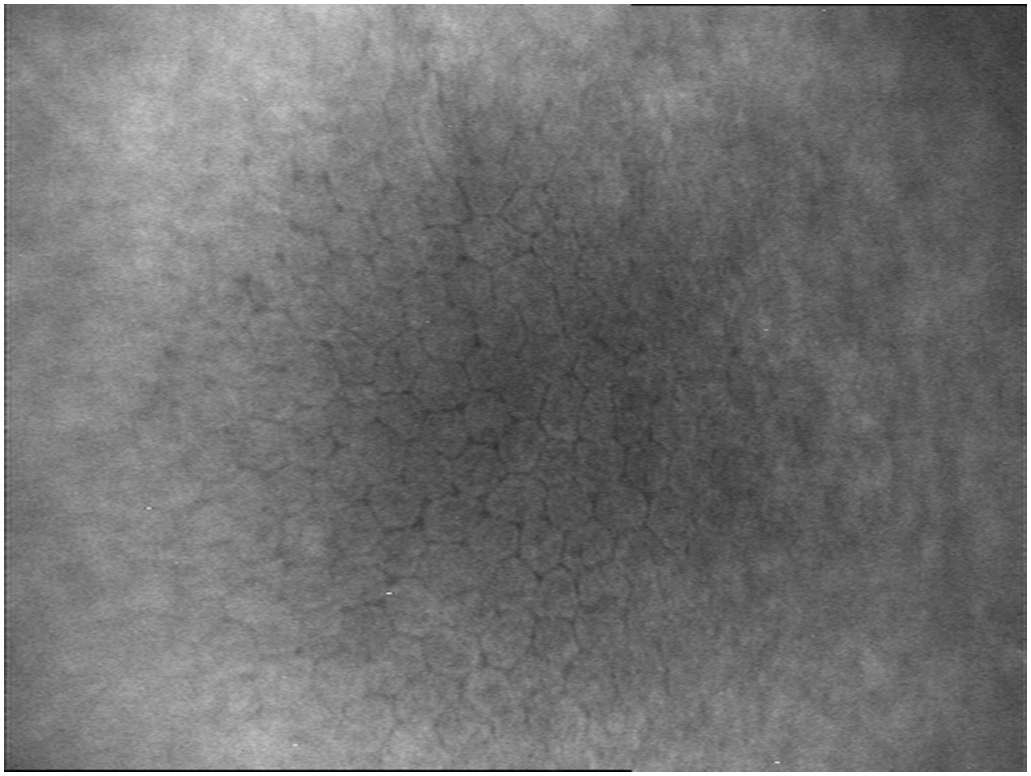

Supplementary Figure 24: Evaluation of the corneal endothelium phenotype by Confoscan4 scanning microscope in cigarette smoke-exposed mouse 24 (CS-24).

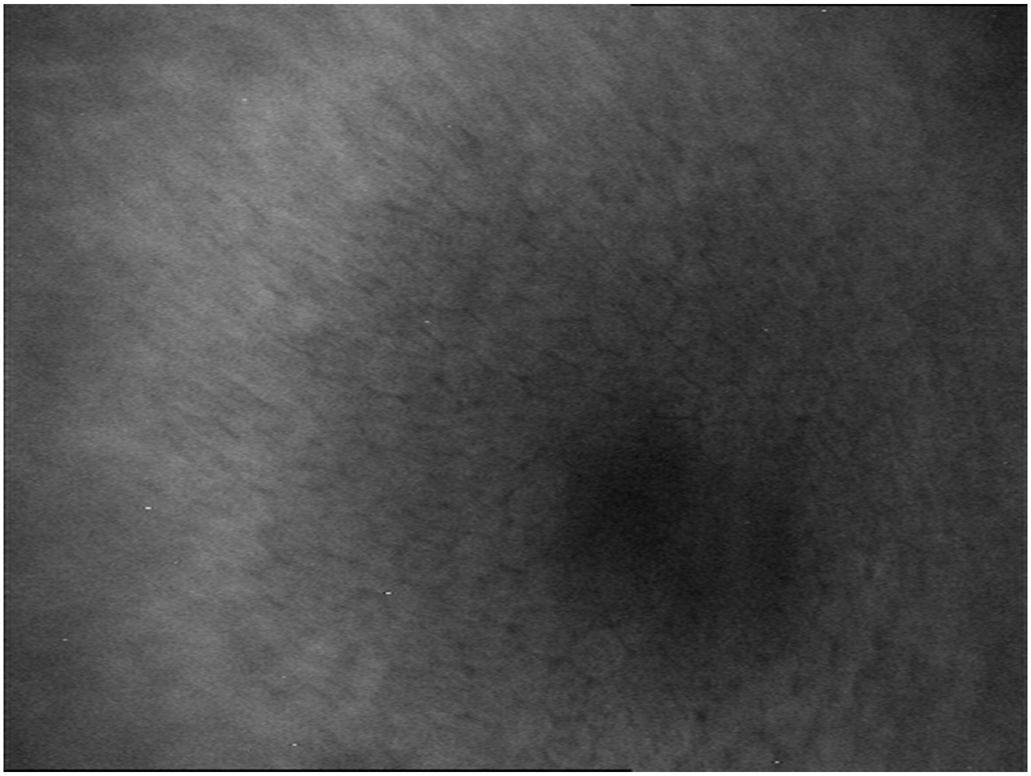

Supplementary Figure 25: Evaluation of the corneal endothelium phenotype by Confoscan4 scanning microscope in cigarette smoke-exposed mouse 25 (CS-25).

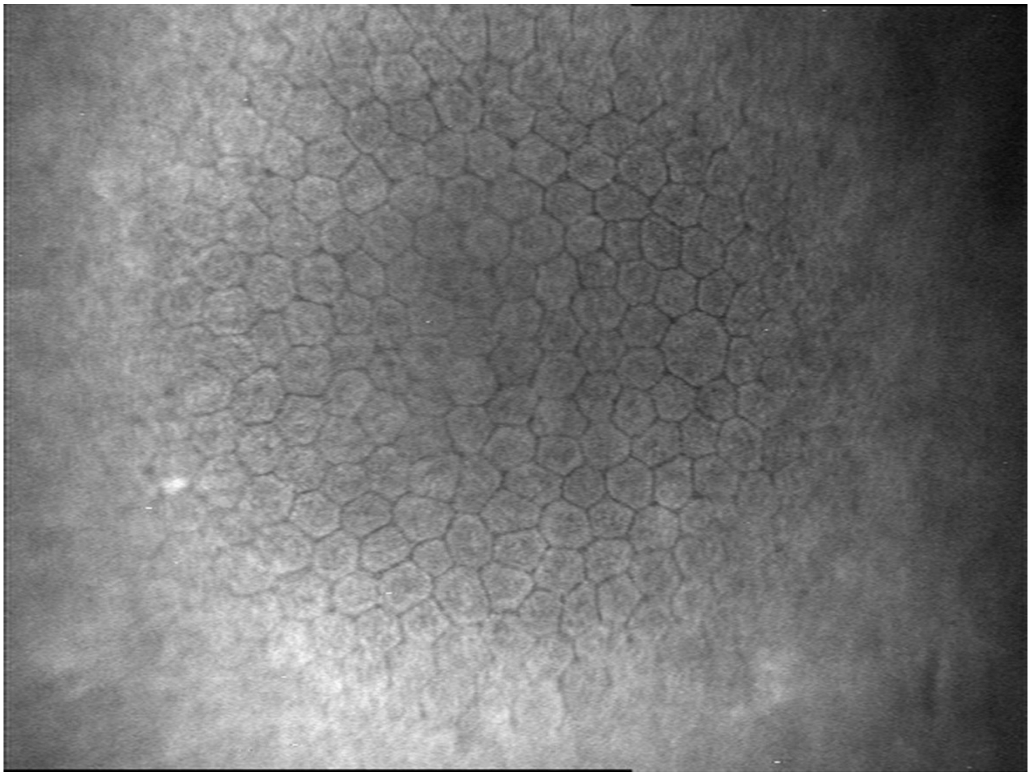

Supplementary Figure 26: Evaluation of the corneal endothelium phenotype by Confoscan4 scanning microscope in cigarette smoke-exposed mouse 26 (CS-26).

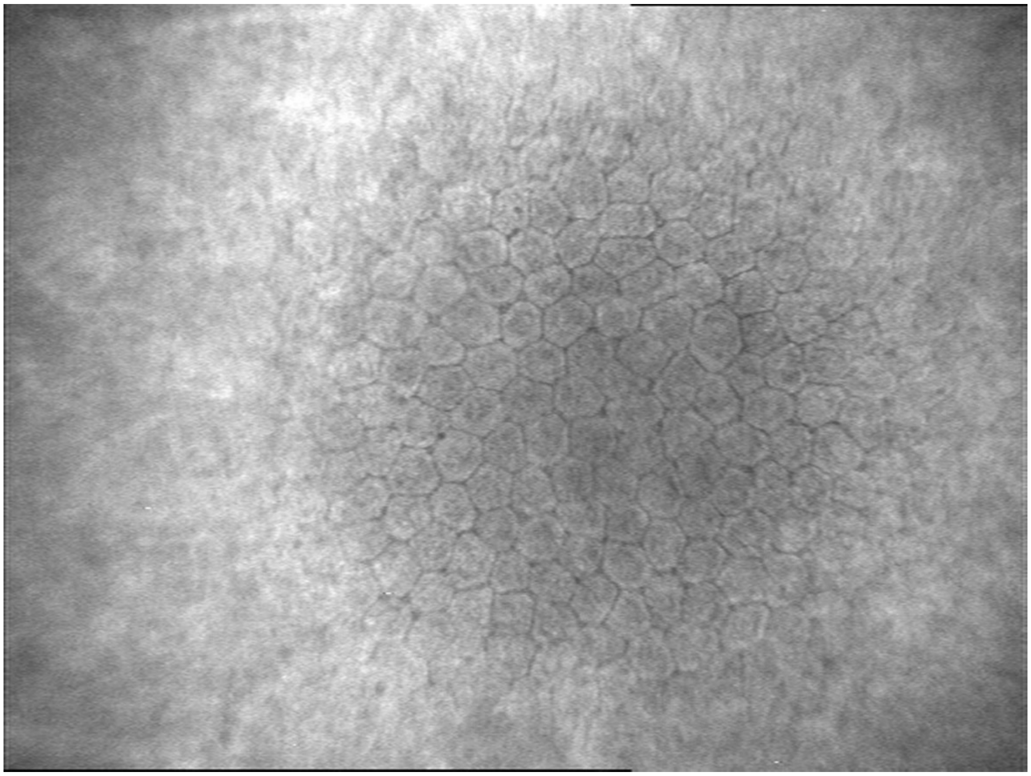

Supplementary Figure 27: Evaluation of the corneal endothelium phenotype by Confoscan4 scanning microscope in cigarette smoke-exposed mouse 27 (CS-27).

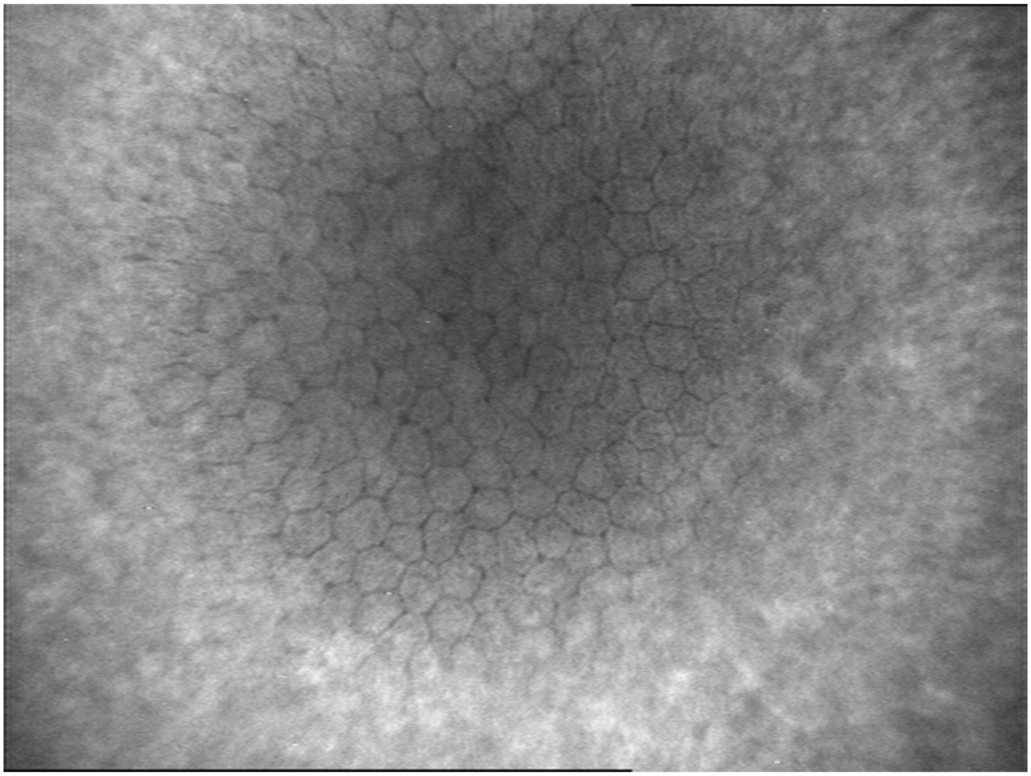

Supplementary Figure 28: Evaluation of the corneal endothelium phenotype by Confoscan4 scanning microscope in cigarette smoke-exposed mouse 28 (CS-28).

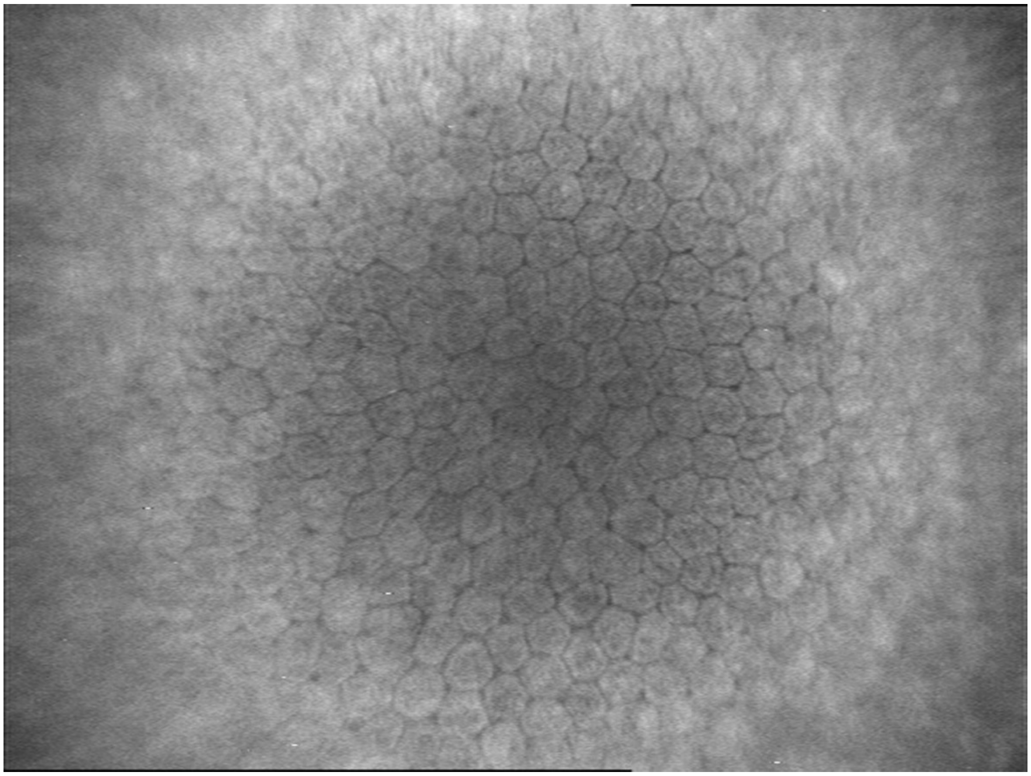

Supplementary Figure 29: Evaluation of the corneal endothelium phenotype by Confoscan4 scanning microscope in cigarette smoke-exposed mouse 29 (CS-29).

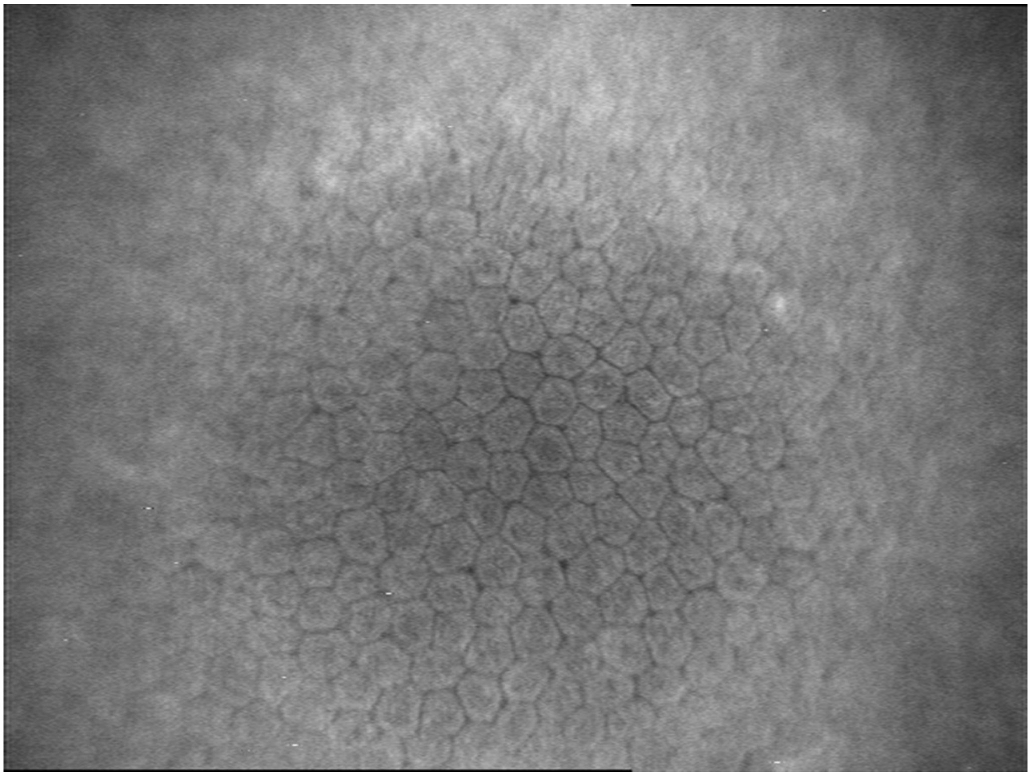

Supplementary Figure 30: Evaluation of the corneal endothelium phenotype by Confoscan4 scanning microscope in cigarette smoke-exposed mouse 30 (CS-30).

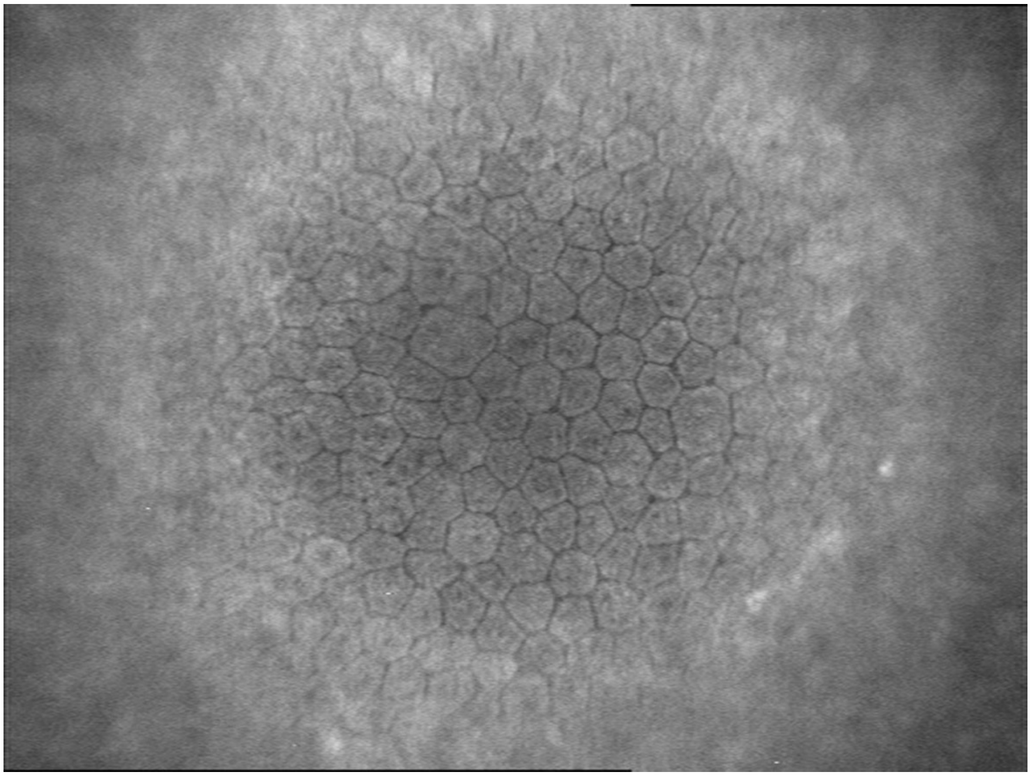

Supplementary Figure 31: Evaluation of the corneal endothelium phenotype by Confoscan4 scanning microscope in cigarette smoke-exposed mouse 31 (CS-31).

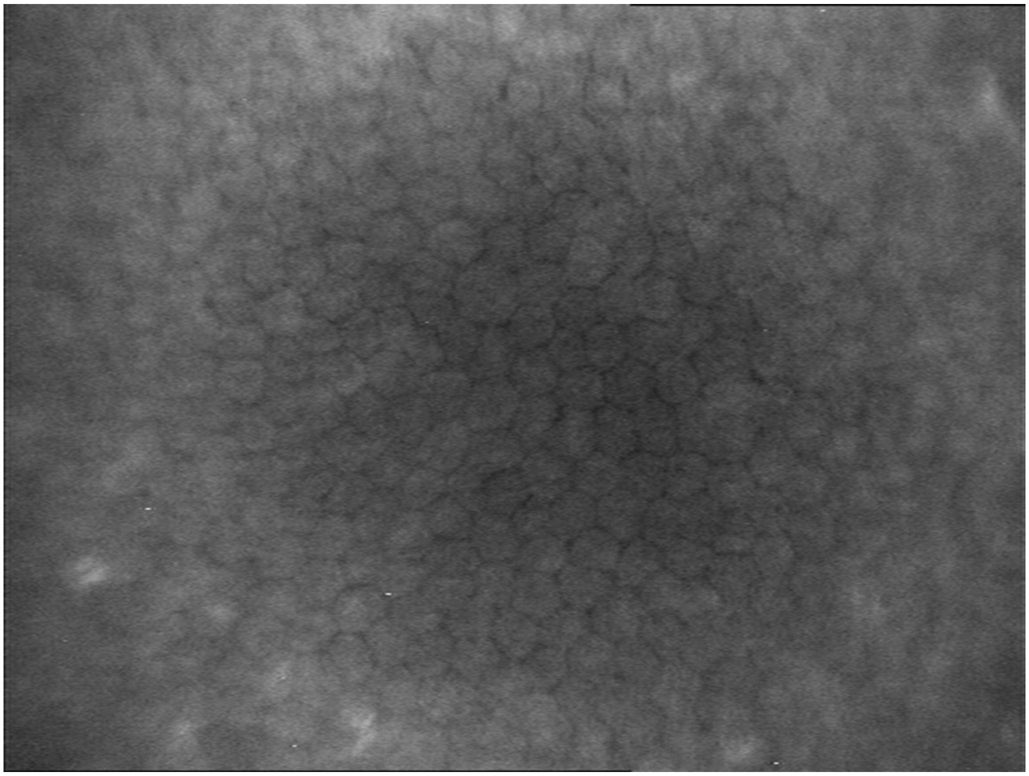

Supplementary Figure 32: Evaluation of the corneal endothelium phenotype by Confoscan4 scanning microscope in cigarette smoke-exposed mouse 32 (CS-32).

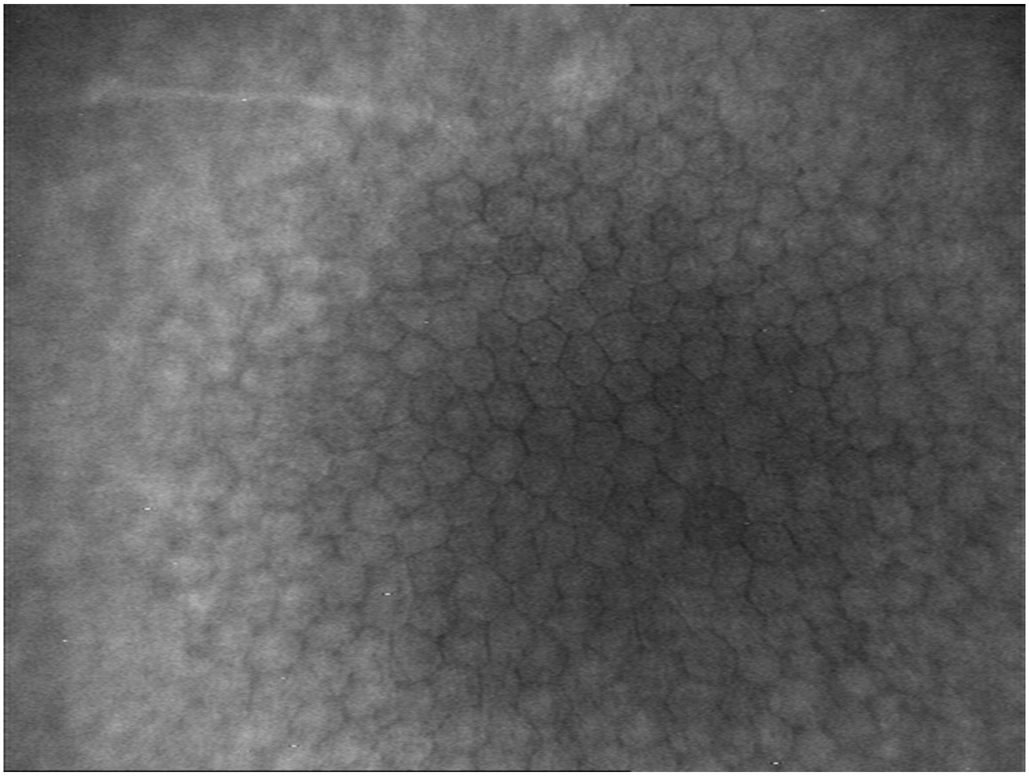

Supplementary Figure 33: Evaluation of the corneal endothelium phenotype by Confoscan4 scanning microscope in control mouse 1 (Ct-1).

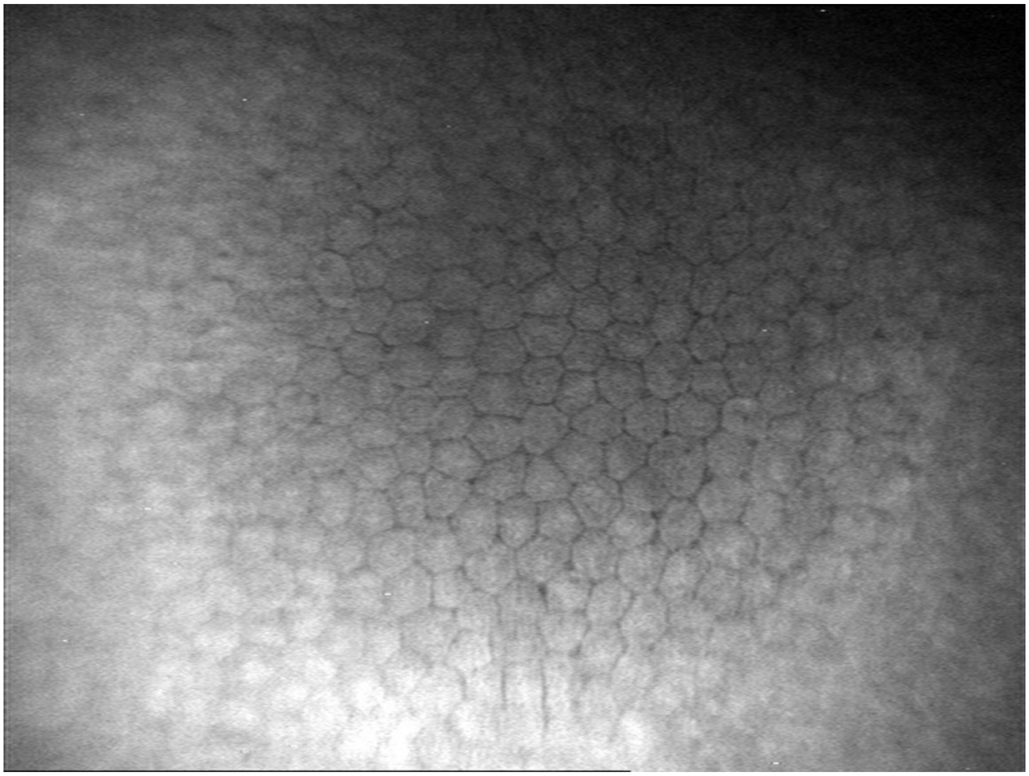

Supplementary Figure 34: Evaluation of the corneal endothelium phenotype by Confoscan4 scanning microscope in control mouse 2 (Ct-2).

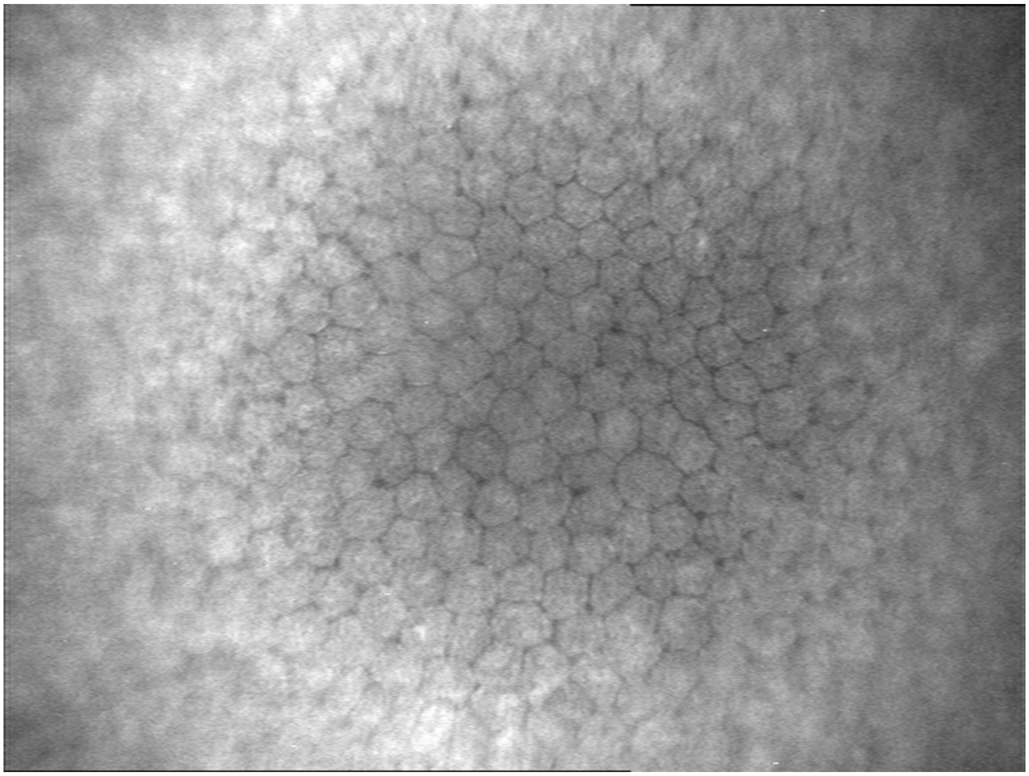

Supplementary Figure 35: Evaluation of the corneal endothelium phenotype by Confoscan4 scanning microscope in control mouse 3 (Ct-3).

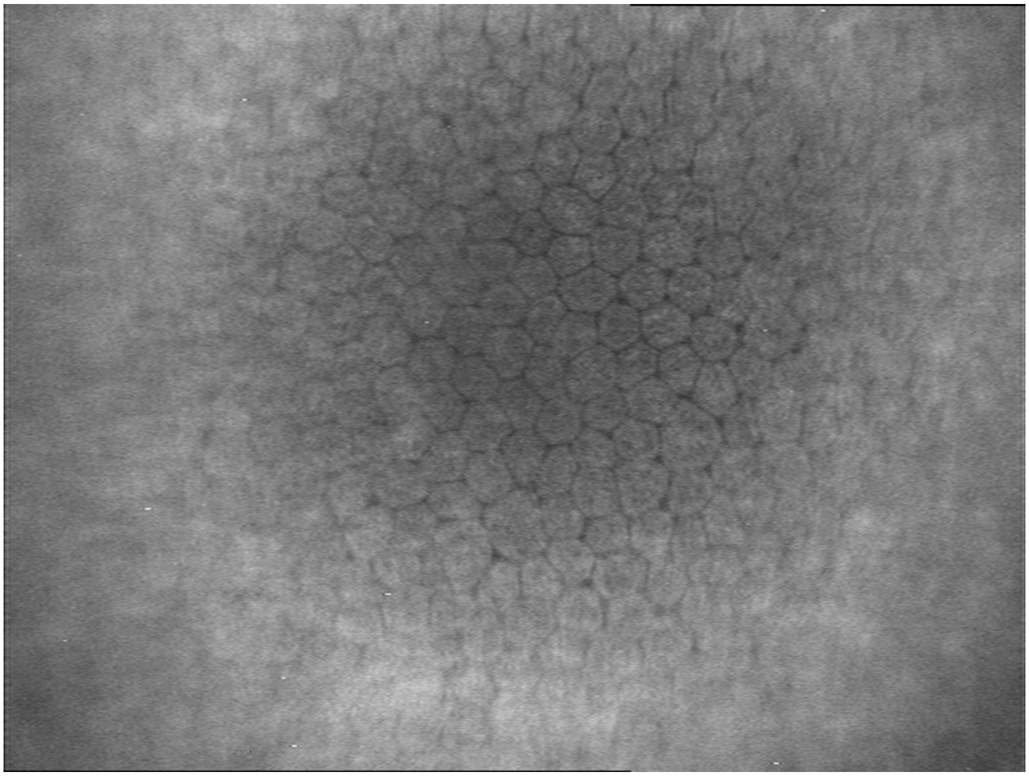

Supplementary Figure 36: Evaluation of the corneal endothelium phenotype by Confoscan4 scanning microscope in control mouse 4 (Ct-4).
